# Digital Health and Machine Learning Technologies for Blood Glucose Monitoring and Management of Gestational Diabetes

**DOI:** 10.1109/RBME.2023.3242261

**Published:** 2023-02-07

**Authors:** Huiqi Y Lu, Xiaorong Ding, Jane E Hirst, Yang Yang, Jenny Yang, Lucy Mackillop, David Clifton

**Keywords:** blood glucose sensors, gestational diabetes, digital health, machine learning, patient monitoring

## Abstract

Innovations in digital health and machine learning are changing the path of clinical health and care. People from different geographical locations and cultural backgrounds can benefit from the mobility of wearable devices and smartphones to monitor their health ubiquitously. This paper focuses on reviewing the digital health and machine learning technologies used in gestational diabetes – a subtype of diabetes that occurs during pregnancy. This paper reviews sensor technologies used in blood glucose monitoring devices, digital health innovations and machine learning models for gestational diabetes monitoring and management, in clinical and commercial settings, and discusses future directions. Despite one in six mothers having gestational diabetes, digital health applications were underdeveloped, especially the techniques that can be deployed in clinical practice. There is an urgent need to (1) develop clinically interpretable machine learning methods for patients with gestational diabetes, assisting health professionals with treatment, monitoring, and risk stratification before, during and after their pregnancies; (2) adapt and develop clinically-proven devices for patient self-management of health and well-being at home settings (“virtual ward” and virtual consultation), thereby improving clinical outcomes by facilitating timely intervention; and (3) ensure innovations are affordable and sustainable for all women with different socioeconomic backgrounds and clinical resources.

## I Introduction

Gestational diabetes mellitus (GDM) is defined by the World Health Organization (WHO) as carbohydrate intolerance resulting in hyperglycemia - high blood glucose - of variable severity with onset or first recognition during pregnancy [1]. It is one of the most common non-communicable medical complications during pregnancy. The prevalence of GDM is increasing rapidly worldwide. In 2021, the average prevalence of GDM was 16.7% globally, and was highest in Southeast Asia at 25.9%, as reported based on the population, the diagnostic criteria used, and geographical locations [2–6]. GDM is associated with both short- and long-term adverse health consequences for mothers and children. During pregnancy, GDM is associated with an increased risk of pre-eclampsia, increased fetal growth leading to macrosomia, shoulder dystocia, birth trauma and neonatal hypoglycaemia [7, 8]. Women with GDM also have a 50% risk of developing type 2 diabetes in the decade following their pregnancy [9, 10]. This makes GDM a condition of great public health interest in the fight against the global epidemic of type 2 diabetes.

During a healthy pregnancy, particularly in the second half of pregnancy, placental hormones favour a state of insulin resistance, resulting in increased insulin secretion from the pancreatic β-cells [11]. There is a higher risk of developing hyperglycaemia for women in whom insulin secretion is inadequate, or those with higher peripheral insulin resistance [10].

The most widely accepted test for diagnosing GDM is the 75-gram oral glucose tolerance test (OGTT), recommended by the WHO [12] and UK National Institute for Health and Care Excellence (NICE) [13]. Using the WHO/IADPSG criteria, GDM is diagnosed if the woman has either a fasting plasma glucose level of 5.1 mmol/L or above, 1-hour ≥10.0 mmol/L, or 2-hour ≥ 8.5 mmol/L. However, these thresholds are not globally agreed. Many counties, including the UK, recommend their own national thresholds to diagnose GDM. This diversity in diagnosis is also reflected in a lack of universal clinical management and monitoring targets.

After diagnosis, glycaemic management in GDM is based on self-collected glucose, most commonly through fingerstick capillary blood testing. Different organizations use different blood glucose targets in women with GDM. For example, NICE 2015 (and updated in 2020) [13–15] recommends targets of fasting < 5.3 mmol/L and 1-hour postprandial <7.8 mmol/L. Women with fasting glucose levels of 7.0 mmol/L or above are advised to start medication treatment immediately. Women with fasting glucose < 7.0 mmol/L are initially given lifestyle advice, such as diet and regular exercise. Diet and medication plans should be reviewed by a clinician every two to four weeks, either during hospital visits, telephone calls or remote-monitoring platforms [16]. If blood glucose targets are not met within 1-2 weeks, women should be offered medication (e.g., metformin or/and insulin) [15, 17]. In comparison, the American Diabetes Association recommends similar glucose targets for women with GDM as follows: fasting glucose less than or equal to 95 mg/dL, 1 hour after eating less than or equal to 140 mg/dL (equivalent to 7.8 mmol/L), and 2 hours after eating less than or equal to 120 mg/dL (equivalent to 8.6 mmol/L) [18].

Due to the vital role of glycaemic monitoring and the changing physiology as pregnancy progresses, women with GDM are asked to monitor their blood glucose levels daily. The monitoring results are generally labelled as fasting pre-meal, one-hour post-meal and at bedtime.

To better support women performing glycaemic monitoring, and to provide clinicians and patients with clinically traceable measures, digital health technologies have been developed for GDM. The U.S. Food and Drug Administration (FDA) defines digital health as mobile health (m-Health), health information technology, wearable devices, telehealth and telemedicine, and personalized medicine [19]. The power of digital innovations and machine learning aims to facilitate the prevention, early diagnosis and management of people’s health in hospital, community and home settings.

**Fig. 1:**
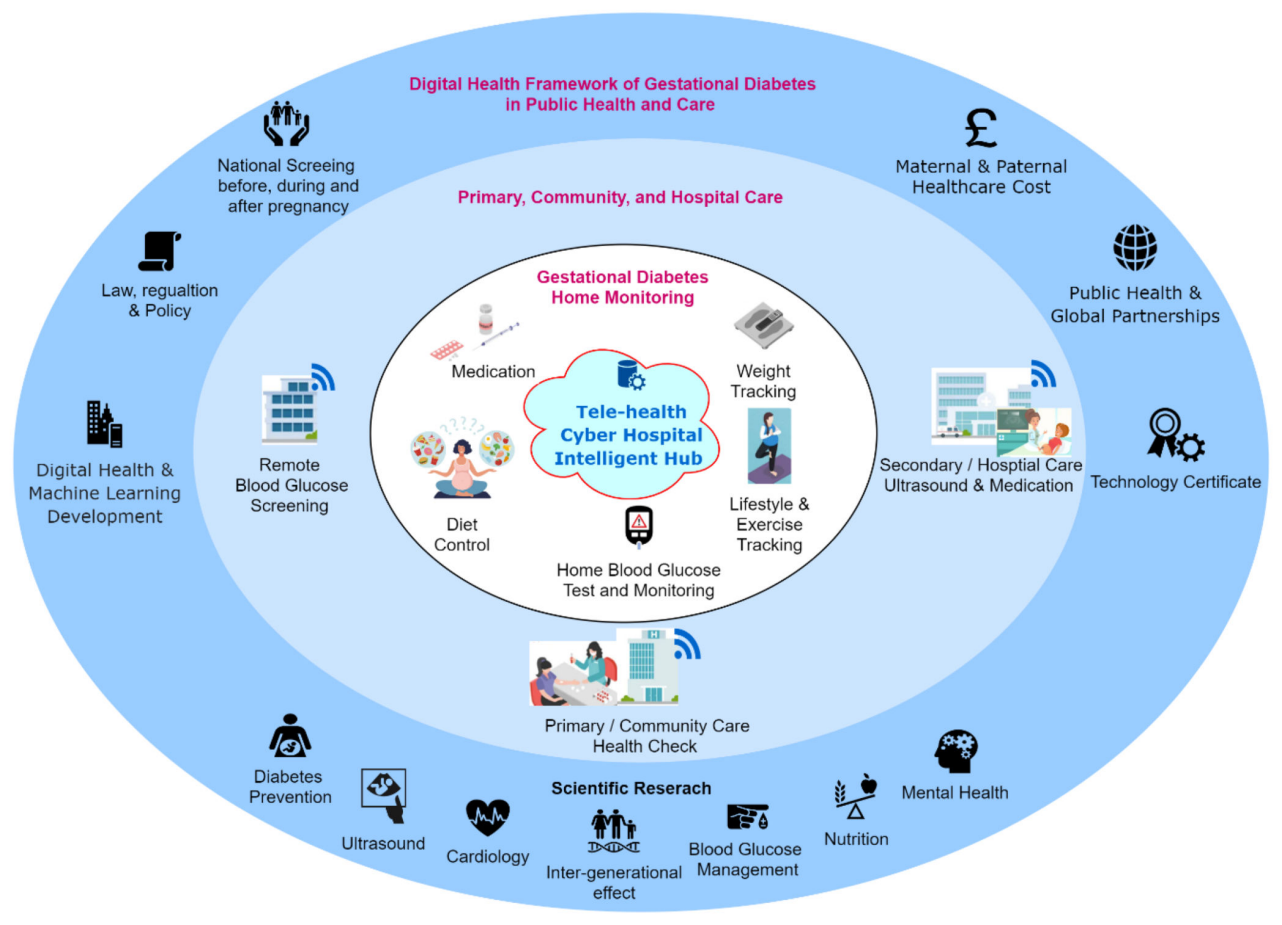
Digital health for antenatal and postnatal health and care in hospital, community and home care environments

Fig. 1 demonstrates potential scenarios of how digital health and machine learning could be fused into the health and care infrastructure for people with gestational diabetes. There are three rings: (1) the dark-blue outer ring demonstrates the components of the digital health framework of gestational diabetes in public health and care, including the maternal and neonatal healthcare cost, public health and global partnerships, law, regulation and policy, digital health innovation, technology certificate, national screening services and scientific research; (2) the light-blue ring shows three healthcare environments, including primary and community care, hospital care as well as the diabetes screening services before and after the pregnancy; and (3) the white inner ring demonstrates the scenarios of personal gestational diabetes self-monitoring at home, including weight tracking, blood glucose self-test and monitoring, diet control and medical interventions, such as metformin pills or insulin injections. These three rings are comprehensive to each other and demonstrate three levels of antenatal and postnatal GDM care infrastructure for patients with GDM. Blood glucose monitoring is often linked to wearable sensors in mobile devices, such as smartphones, watches, and waistbands. The ability to collect, collate and analyse data from different sensors for activity tracking, food intake quantification, blood glucose monitoring and medication management could open up new possibilities to help people manage GDM. But there is a gap for data scientists, engineers and clinicians to fill. Personalized, explainable, and trusted AI and ML models are needed to assist patients and clinicians in clinical management with the aim of improving patients’ lifestyles and short-term and long-term clinical outcomes.

For conditions such as GDM, digital health technologies can provide real-time glucose monitoring, thus enabling timely diagnosis, treatment and personalised advice on food intake, exercise and medication. Current methods for glucose measurement in women with GDM only provide a snapshot of the change in glucose levels. Ideally, glucose levels should be monitored in real-time, using a non-invasive and unobtrusive method, such as continuous glucose monitoring.

A review on gestational diabetes by Saravanan [6] suggests that a shift is needed to move from the perception of a short-term condition that confers an increased risk of large babies to a potentially modifiable long-term condition that contributes to the growing burden of childhood obesity and cardio-metabolic disorders in women and their offspring.

In this paper, we provide comprehensive reviews of (i) glucose monitoring technologies for women with GDM; (ii) the types of glucose sensor technologies currently available and their potential for application in GDM; and (iii) digital health technologies and machine learning algorithms for blood glucose prediction, medication advice and lifestyle management in GDM, with comparison to the current state-of-the-art technologies used in type 1 and type 2 diabetes monitoring. We then finally discussed potential future work in this field to improve the health and care of women with GDM.

## II Blood Glucose Monitoring Techniques for Gestational Diabetes

Blood glucose monitoring plays a vital role in the early detection, diagnosis, treatment and management of women who have gestational diabetes. Health providers use blood glucose values to check and adjust the effect of treatments, such as the effectiveness of diet and exercise and the dosage of medications. There are two main methods for ambulatory glycemic testing: intermittent capillary blood glucose technologies and continuous glucose monitoring technologies (CGM). CGM can be subdivided into intermittently scanned (flash) and real-time continuous (CGM) sensors.

These two kinds of sensors are different in four aspects: i) intermittent blood glucose monitoring measures discrete glucose levels accurately from capillary blood, whereas continuous monitoring provides multiple glucose levels of fair accuracy from the interstitial fluid beneath the skin, which approximates blood glucose levels; ii) with standard intermittent monitoring, current blood glucose levels do not predict future glucose levels; but with continuous monitoring, trends in glucose levels are often predicted to enable the insulin pump to provide a precise amount of insulin accordingly; iii) with intermittent monitoring, blood glucose results can be used directly without data processing; but with continuous monitoring, data analysis is required to extract fluctuations of GL, for example, mean blood glucose results at different events, such as before or after breakfast; and iv) an intermittent blood glucose monitor requires patient’s input for every reading, whereas a continuous monitor records the time-series blood glucose value synchronously.

It is challenging for patients to self-monitor their blood glucose using fingerstick testing four to six times a day; however, this is especially important for women who are on multiple daily insulin injections to balance the risk of hyperglycaemia and hypoglycaemia. Finger stick testing is inconvenient and painful for women, with limitations including accuracy, specificity, and inappropriate usage. Because of cost and lack of evidence, continuous blood glucose monitoring is an alternative, but it is not used broadly in clinical practice for gestational diabetes management.

This section will explain the technical aspects of intermittent and continuous sensor technologies in detail.

### A Periodic Monitoring using Fingertip Blood Tests

**Fig. 2:**
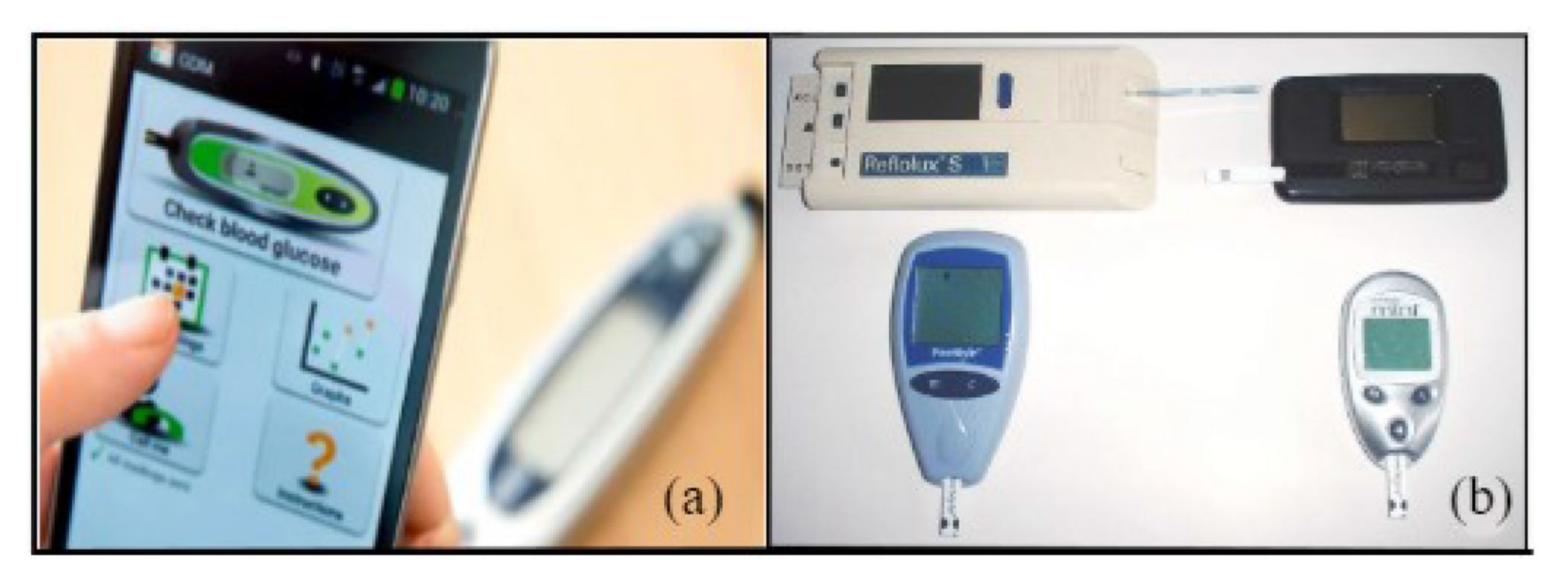
Self-monitoring glucose meters (a) fingertip blood glucose testing with mobile connections [20, 21], (b) four generations of blood glucose meters (c. 1987-2005): Top left: Reflolux S (Accu-Chek III in the U.S.), by Boehringer Mannheim, 2-minute read time, based on reflectance; top right: ExacTech Card, by MediSense, 30-second read time, electrochemical test stripe; bottom left: FreeStyle, by TheraSense, 15-second read time, electrochemical test stripe; bottom right: Freestyle Mini, by Abbott, 7-second test time, electrochemical test stripe. [22].

In order to gain an accurate picture of blood glucose trends, women in pregnancy are requested to perform several fingerstick tests each day. The test is typically performed by piercing the skin – usually on the fingertip – with a lancet device to obtain a small volume of blood, and then the glucose concentration is determined with a glucose meter (Fig. 2a). The development of the first blood glucose meter dates back to the 1970s after developments in urinary glucose testing and blood glucose dry-reagent test strips [23, 24]. The first blood glucose meters combined dry chemistry test strips (Dextrostix) with reflectance photometry to measure blood glucose, of which the paper strip is treated with enzyme reagents that give approximate results for glucose concentration with one drop of whole capillary or venous blood. Since then, significant progress has been achieved in the development of blood glucose meters, such as reducing blood sample size, obtaining blood samples from alternate sites, and improving test time, display, data storage, and calibration (2b) [23, 25]. Nowadays, there are a large number of devices on the market, with meters developed by Roche Diagnostics, Lifescan, Medisense, Bayer, and Therasense among the most popular [25].

Though popular and easy to use, self-monitoring of blood glucose using finger-stick test strips has limitations, including accuracy, specificity, and inappropriate usage, as discussed by Olansky et al. [26]. To be safe and of clinical value, blood glucose meters should measure blood glucose levels accurately and precisely. In terms of accuracy, the standard developed by the FDA in 2016 for blood glucose meters for over-the-counter use requires 95% of data pairs of BGM measurement and a reference measurement to be within 15% for BG values >100 mg/dL (5.55 mmol/L), which is similar to the standard issued by the International Organization for Standardization (ISO) 15197:2013 [27, 28].

### B Continuous Blood Glucose Monitoring using Wearable Sensors

With advances and the development of technologies in electronics, manufacturing, materials and sensors, glucose monitoring has developed rapidly. Technology enabling accurate, continuous, long-term and noninvasive glucose monitoring has become possible, with blood no longer the only medium for glucose monitoring.

Compared with finger-stick blood testing, continuous glucose monitoring (CGM) – either from real-time use or intermittently viewed – provides insights about the direction, magnitude, duration, frequency, and fluctuations in glucose levels, which enables sufficient information that is clinically valuable [29]. CGM can reduce risks of hypoglycemia, hyperglycemia, and glycemic variability and improve the quality of life for patients with blood glucose imparity, including patients with gestational diabetes. CGM via an implantable, transdermal sensor has become the gold standard method for monitoring continuous glucose levels in pregnant women with type 1 diabetes. CGM has been widely studied and is now being used in clinical settings [30]. However, prolonged use of CGM is invasive and may cause vascular damage or infection [31]. No articles have been published on their long-term performance.

The evidence for CGM use in pregnancy comes mostly from women with type 1 diabetes, and in the UK, the technology is currently not recommended for women with other forms of diabetes [17]. There are several barriers that will need to be addressed before CGM can be considered for women with GDM. The first barrier is cost. Depending on the length of time it is used, real-time CGM can cost up to £2000 per pregnancy (2021 NHS list price). With up to 65000 women having GDM in the UK each year, the potential cost increase of adopting CGM for GDM could be > £100 M per year. The second barrier is the limited understanding of managing GDM using glycaemic time in range. The optimal time in range for women with GDM is unknown [29]. Finally, it is unclear whether outcomes would be improved for women and their babies with GDM by adopting CGM; thus, adequately powered clinical trials are needed in this area.

However, given the trend of reducing the cost of technologies, ongoing technological developments and research in this area, it is possible in the future that CGM may become an option for women with GDM. The remainder of this section of this review will focus on noninvasive continuous glucose monitoring techniques with the potential for application in GDM.

Noninvasive continuous glucose sensing can be classified into three categories: electrochemical methods, optical methods, and non-optical methods, including microwave methods. Each of these technologies will be elaborated as below.

#### a) Enzyme-based electrochemical sensors

**Fig. 3:**
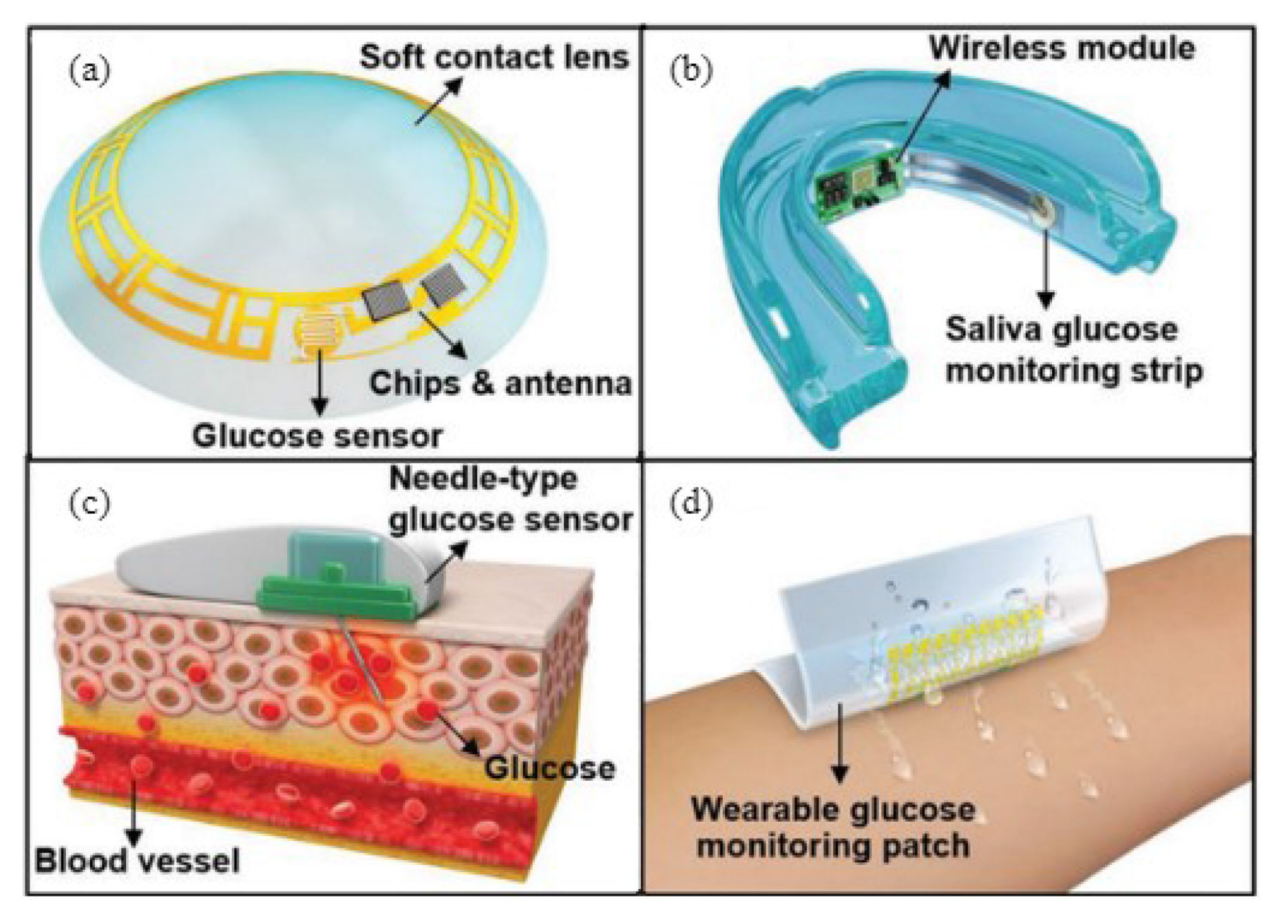
Noninvasive enzyme-based glucose monitoring sensing systems through different contact agents and body sensors (a) contact lens glucose sensor on tear, (b) saliva glucose monitoring strip on saliva, (3) needle-type glucose sensor on insulin sensitivity factor (ISF), and (d) wearable glucose monitoring patch on sweat. [33]

Enzyme-based electrochemical sensing combines enzymatic detection with electrochemical measurements, and it can yield higher detection accuracy than each of the single techniques. The enzymatic sensor is performed on bio-fluids that contain lower glucose concentrations than blood, for example, interstitial fluid, tears, saliva, and sweat [32] (Fig. 3). For example, the well-known Google contact lens is such a sensor, designed to detect glucose levels in tears. Alternatively, interstitial fluid has been used as the medium to extract glucose onto the skin surface by using the technique of reverse iontophoresis with an enzymatic glucose sensor. GlucoWatch® (Cygnus Inc., Redwood, CA, USA) is a commercial product that adopted reverse iontophoresis as the way to extract glucose samples.

One of the main advantages of such technology is that it is noninvasive, unobtrusive, and real-time. This technology can be realized via flexible epidermal sensors that can be fabricated into body-compliant wearable platforms, such as a patch, wrist band, temporary tattoo, and integrated wireless electronics for practical wearable applications [34].

However, there are two disadvantages of such methods. One is that there is a time lag between the blood glucose level and the glucose in interstitial fluid; the other is that such a method by applying potential onto the human skin (or other epithelial surfaces) can cause irritation [35]. The latest research and development of epidermal electrochemical glucose sensors can be found in more detail in the review paper by Kim et al. [34]. A more elaborative review of enzyme-based electrochemical glucose sensors, including materials, device structures, fabrication processes, and system engineering, can be found in the review by Lee [33].

#### b) Methods based on optical detection

Unlike electrochemical methods, physical approaches to detect glucose levels are based on measuring specific properties of the glucose molecule. Among the physical approaches, the optical sensing method is one of the most common methods for CGM due to its potential benefits of increased stability and low [36]. The optical approaches mainly include infrared (IR) spectroscopy, Raman spectroscopy, photoacoustic spectroscopy, fluorescence spectroscopy, and optical coherence tomography (OCT) [37, 38]. The following sections will give an overview of how these technologies are used in continuous glucose sensing.

IR spectroscopy usually consists of near-infrared (NIR) and mid-infrared (MIR) spectroscopy depending on the light wavelength – the short-band 780-1500 nm and 4000-400 cm-1 ranges are usually used for NIR and MIR, respectively. For noninvasive studies, reflectance light-emitting diode (LED) arrays that are readily available and low-cost are commonly used for NIR as the sources, and the sensing location can be fingertip, forearm or upper arm. For MIR, glucose has absorption peaks in several regions, and the 1200-1000 cm-1 range has received the most attention in sensor studies because it is related to the skeletal vibrations of glucose. Compared with NIR absorption bands that are typically combined bands, weaker and broader, the MIR range absorption bands are relatively sharp, more selective and have a stronger signal. For IR spectroscopy, the common challenge is the interfering molecules that have absorption spectra similar to glucose; examples of these molecules include lactate, urea, and sugars.

Raman spectroscopy, as its name indicates, employs Raman scattering in order to observe vibrational modes in glucose molecules [39]. Usually, a single-wavelength source with visible or NIR wavelengths is sufficient to produce the entire Raman spectrum, as only the frequency shift is measured. The resulting frequency shift due to the scattering is sensitive to the vibrational modes of the molecule and independent of the excitation photon frequency. And therefore, the Raman spectrum for glucose can be quite clearly distinguished from other biological compounds. Furthermore, the Raman signal can be amplified by several orders of magnitude with the technique of surface-enhanced Raman scattering (SERS). However, the main challenge of Raman spectroscopy is the small cross-section, which can be ten orders of magnitude smaller than the fluorescence cross-section, resulting in the Raman scattering signal being masked by interfering fluorescence signals.

As mentioned above, both IR and Raman spectroscopy detect glucose concentration through the direct interaction between light and glucose. In contrast, fluorescence sensing does not measure glucose directly but measures the signal from molecules that can reversely bind to glucose. These molecules are called exogenous fluorophores; they are engineered to form a complex with glucose molecules and only fluoresce in the presence of glucose. As a result, the fluorescent light intensity will depend on the glucose concentration, since more fluorophores are active when there is more glucose bound with them.

OCT is a measurement method that uses an interferometer with low coherence light – typically light in the NIR range due to its nature of miniaturisation and low cost. The OCT system consists of one reference and one sample arm for the light, a moving window to vary the path length, and a photodetector for the light. The light scattered back from the tissue is combined with light from the reference arm, and the interference signal is sent to the photodetector. The reflective index of the interstitial fluid will change when the glucose concentration changes, which in turn changes the scattering coefficient. This change in scattering coefficient and concomitant variation in the interferogram is used to determine the glucose concentration. The main challenges of OCT for glucose monitoring are that the measured change in the scattering coefficient is small and sensitive to motion artefacts.

#### c) Other noninvasive glucose detection technologies

**Fig. 4:**
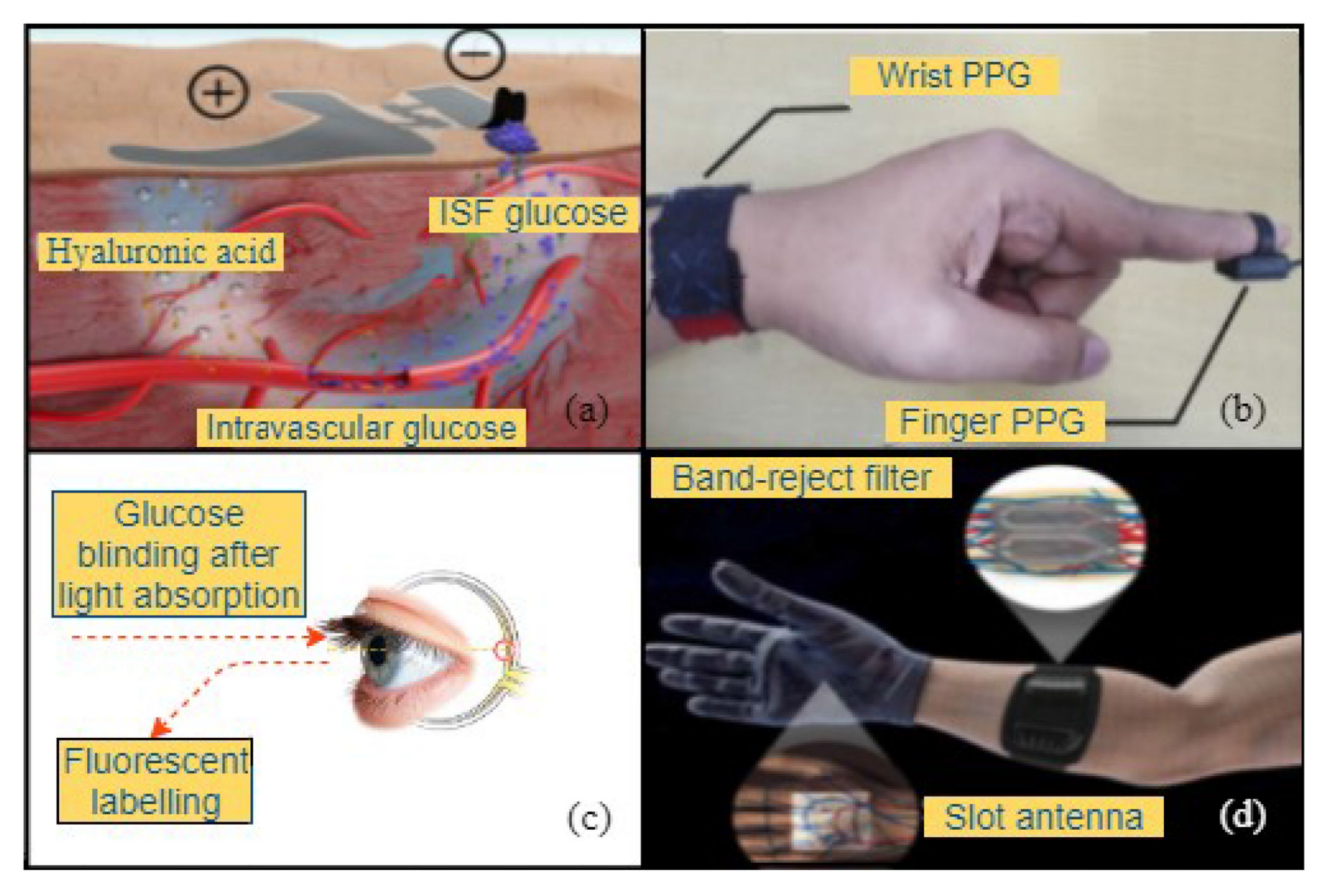
Noninvasive continuous glucose sensing techniques (a) skin-like glucose biosensor [41], (b) wearable-band type near infrared (NIR) optical biosensor [42], (c) sensing through fluorescent labelling [38], and (d) microwave sensors [40].

Except for the commonly studied optical sensing approaches, other noninvasive methods, such as electrical impedance spectroscopy and microwave sensing methods, have also been explored, as shown in Fig. 4. The electrical impedance spectroscopy (EIS) method has the advantages of being low-cost and user-friendly. The design of such systems consists of bioimpedance sensors, signal measurement strategies, modelling and parameter estimation methods to extract blood glucose levels, and portable system designs [35]. While microwave technology measures the dielectric properties of aqueous glucose with a microwave sensor, generally, a patch antenna which is compact, cost-effective, painless and has the potential to provide a more accurate measurement. For example, inspired by vasculature anatomy topologies, Hanna et al. developed a tunable electromagnetic multi-sensing system that is noninvasive and wearable for continuous glucose monitoring [40]. Such systems achieved a high correlation between system’s physical parameters and blood glucose levels [36, 37].

In general, the advantages of optical and microwave methods lie in their highly non-invasive nature and continuous monitoring without stimulating discomfort to the human body. However, the measured value may not be highly correlated with the actual blood glucose value as the linear range is narrow, so subsequent algorithm correction is required. More detailed information on various sensing technologies for glucose monitoring can also be found in the reviewer papers on continuous blood glucose monitoring [36, 37].

## III Emerging Monitoring Devices and Management Platforms for Gestational Diabetes

Based on the periodic fingerstick and continuous blood glucose monitoring technologies introduced in Section II, various platforms for monitoring and managing gestational diabetes have been implemented globally in both clinical and non-clinical applications, and in either commercial or research settings. In this section, we will review a few exemplary ones that started to make an impact on the digital health industry for gestational diabetes.

### A Devices and Applications Trailed for Clinical Observations and Interventions

The NIH ClinicalTrials.gov is a database of privately and publicly funded clinical studies conducted worldwide, managed by the NIH U.S. National Library of Medicine. To provide insight into digital health technologies used in clinical practices worldwide, we screened 538 studies (accessed on 8 August 2021) using the keyword “gestational diabetes” on the NIH ClinicalTrials.gov database. After shortlisting clinical trials using keywords, and removing clinical trials that were not completed or not started, we shortlisted 16 observational studies and 36 interventional studies that used digital health and mobile health technologies for patient monitoring. Protocols were reviewed under two categories: observational studies and clinical interventions.

People living with GDM require self-management to maintain blood glucose levels, including diet and exercise control, as well as through medication intervention. Table 1 summarises the technologies in the primary purpose, outcome measures, monitoring method, patient size, country of use, and completion status. These studies focus on the technologies for blood glucose monitoring, medical intervention, and lifestyle management for weight control. Readers interested in clinical trial details can read the full details of the above trials at the NIH ClinicalTrials.gov. Systematic clinical reviews in gestational diabetes monitoring systemic clinical reviews [4, 43–45] and studies on clinical outcomes [46, 47] are also helpful reading materials. Countries with different prevalence levels of GDM, limitations in doctor resources, or different income levels, such as low- and-medium income countries, often require cost-effective and different digital health solutions to address their challenges. Country-specific reviews [3, 48, 49] can provide a comprehensive source of information that addresses risk factors, diagnosis criteria in racial variance and geographical differences that suit epidemiology studies.

Among the clinical trials listed in Table 1, a few studies have been commercialized and put into clinical practice. For example, in the UK, “*GDm-Health*”, was developed from 2014 -16 [50] and subsequently licensed to Sensyne Health plc in 2017. In a randomised controlled trial, "TREAT-GDm", GDm-Health was deployed on Android mobile phones that can link to Bluetooth-enabled blood glucose meters. To use this self-monitoring platform, patients need to tag their capillary blood readings into six mealtime tags (pre- and post- breakfast, lunch and dinner) and enter their medication dose if applicable. Collected blood glucose measurements are then sent to the GDm-Health server by mobile network and made visible to clinicians. Feedback is provided in the form of colour coding of individual readings: red for high, green for target, and blue for low blood glucose measurements. Summaries of blood glucose measurements (tabulated and graphical) and reminders are also provided as appropriate. The above work has been published [13, 16, 51–54] with a technology appraisal on the NICE website [50]. This innovation has been approved for clinical usage by NICE. As of 2021, GDm-Health is available in 56 Trusts, representing 47% of NHS Trusts in England.

MobiGuide [55, 56] is an evidence-based decision-support system developed in 2014 and then modified in 2017. It can provide personalised decision support based on patients' personal health records, including data from hospital medical records, mobile biosensors, data entered by patients, and recommendations and abstractions output by MobiGuide. This system can be used by patients with gestational diabetes (via mobile MobiGuide) and their care provider (via central MobiGuide). Their study analyzed usage patterns and opinions collected via questionnaires of the 10 atrial fibrillation and 20 (GDM patients and their care providers. The results confirmed using the MobiGuide system have resulted in diagnosis changes for 2/10 atrial fibrillation patients and anticipated changes in therapy for 11/20 GDM patients.

The Pregnant+ smartphone application was developed in 2015 to motivate women to have a healthy diet, and be physically active [57]. Similar to GDm-Health, it allows the automatic transfer of blood glucose measures from the glucose meter to the smartphone. A green or red face indicates a normal or high blood glucose level (hyperglycemia). There is no colour code for the low blood glucose level (hypoglycemia). The trial protocol was published in 2017 [58]. In the trial, women answered questionnaires during pregnancy and were followed up three months postpartum. Trial results were published in 2018 and 2019 [59, 60]. Results suggested that using the Pregnant+ app did not affect the 2-hour glucose level at routine postpartum OGTT. And after controlling for parity, the difference in the emergency caesarean section was not statistically significant.

Apart from blood glucose monitoring, several studies focus on lifestyle interventions, such as diet and physical activity control. The Institute of Medicine guidelines for gestational weight gain are based on pre-pregnancy BMI. Close attention to food intake is necessary during pregnancy to avoid excessive gestational weight gain while ensuring strict glycemic control. Approximately 80% of women with GDM can reach their glycemic goals with diet and lifestyle modiﬁcations alone. Lifestyle interventions are helpful in weight control and may impact pregnancy outcomes.

The behavioural lifestyle intervention (PEARS) RCT in a lifestyle intervention study for women overweight and obesity [61, 62]. A total of 278 pregnant women (BMI 25–39.9 kg/m2) were randomized to the intervention (n = 278), or a control group (n = 287). Results showed that there were no differences between the groups at baseline. Compared with the control group, the intervention group had improved dietary intake post-intervention. Physical activity (MET-minutes/week) was higher in the intervention group post-intervention. App use was associated with a lower glycaemic index and less energy from free sugars, but not with physical activity.

The Mobile-Based Lifestyle Intervention in Women with Glucose Intolerance after Gestational Diabetes Mellitus (MELINDA) study is a Belgian multi-centre RCT in seven hospitals (236 women).[63] The aims of this study are: (1) to evaluate the prevalence and risk factors of glucose intolerance after a recent history of GDM; and (2) to evaluate the efficacy and feasibility of a telephone- and mobile-based lifestyle intervention in women with glucose intolerance after GDM. Women in the intervention group will receive a blended program based on one face-to-face education session and further follow-up through a mobile application and monthly telephone advice. Women in the control group will receive follow-up as in normal routine with referral to primary care. Participants will receive an OGTT one year after baseline. The primary endpoint is the frequency of weight goal achievement (≥5% weight loss if pre-pregnancy BMI ≥ 25 Kg/m^2^ or return to pre-gravid weight if BMI < 25 Kg/m^2^). At each visit, blood samples are collected, anthropometric measurements are obtained, and self-administered questionnaires are completed. Recruitment began in May 2019 and expect to finish in June 2022.

Electronic Monitoring Of Mom’s Schedule (eMOMs) platforms have three clinical studies in Finland. The trial protocol [64] addressed a feasibility randomized controlled study for women with high pre-pregnancy BMI to improve postpartum weight, blood sugar, and breastfeeding. A total of 72 women were included, 24 per group. The design of this trial has combined breastfeeding and the National Diabetes Prevention Program. eMOMS compares the feasibility and efficacy of three interventions on six-month post-partum weight loss among women with a BMI >=25. Patients were recruited at two clinical sites (rural and urban). Program costs will be compared to that of traditionally scheduled group meetings. The study was completed in October 2021, but the results were not published.

Hola Babe and GlucoseMama are the two products that have been used in random clinical trials. The results of these trials were not published, but product information is available on their website for products.

All the above platforms are based on the fingerstick blood test and a telemedicine framework. Chen’s study [65] used CGM on 57 women with GDM, 47 in Israel and ten in California. Data derived from the MiniMed CGM System were compared to fingerstick glucose measurements (6–8 times a day) in 72-hour windows. The time of food intake, insulin injections and hypoglycemic events are recorded in the system and used to monitor the health status of GDM. The study authors suggested that CGM is helpful for adjusting diabetes therapy and can accurately detect high blood glucose and hypoglycemic events that may go unrecognized by intermittent blood glucose monitoring.

### B Consumer-driven Devices and Applications

Along with devices and platforms for clinical purposes, there is a broader commercial market for devices and applications for non-clinical purposes. This section reviews the consumer-level commercial devices and applications used for diabetes. We used the keyword of gestational diabetes to search top-ranked Apps run on iOS using an online search engine (www.deepaso.com). The search date is 18^th^ November 2021, and the results were compared to 18^th^ May 2021. We limited our search to only English and regions with a high prevalence of GDM [66]. The overlap of regions with high prevalence and country-specific ranking data in the search engine are Singapore (SGP), United Arab Emirates (UAE), Thailand (THAI), Malaysia (MAL), United Kingdom (UK), Belgium (BEL) and India (IND).

As shown in Table 2, we listed the top 10 apps across these regions under three categories: Medical (M), Food & Drink (FD), Health & Fitness (HF), and lifestyle (LS). It is noted that the ranking is dynamic and can be misleading when an app is promoted at the searching date. Additionally, the search results included Apps designed for both GDM and general diabetes. The devices and apps that are applicable for GDM overlap with those for diabetes management.

This review lists the top 10 Apps in three categories listed in these seven countries. Several apps in the medical category are ranked high across different counties, such as mySugr- Diabetes Tracker Log, One Touch Reveal, Glucose Buddy Diabetes Tracker, Medisage Phill Reminder, Glucose – Blood Sugar racker, and MySweetGestation. A few of the FD or LS category Apps are ranked high across these seven countries. Within the Medical and Health & Fitness categories, the main features of apps include (i) glucose monitoring, (ii) exercise monitoring or advice, (iii) food intake recommendation and (iii) medicine-taking reminders.

Arguably, the App from the non-medical category is consistently ranked high in different countries and could be popular across these countries.Whereas the Apps belonging to the medical category can be country-specific because of the nature of their development.

The mobile device applications primarily focus on glucose monitoring, exercise tracking, food intake recording, and medication reminders.

#### Blood glucose monitoring

As discussed in section II, consumer-level glucose monitoring uses capillary blood glucose meters or CGM systems. The former requires a user to prick their finger to release a small amount of blood and then place the blood on a test strip readable by the meter. It usually requires the user to manually input those readings in the App, although meters with Bluetooth and NFC are also available. These systems are often associated with reminder features. In contrast, CGM systems can bundle the CGM sensor, smartphone, and smartwatch together. Most existing apps for diabetes management target type 2 diabetes. Some Apps also offer advanced features based on blood glucose levels, including setting alerts for high and low glucose and predicting future glucose levels.

#### Exercise trackers

These are used to record the user’s exercise and other physical activity. Some apps sync the third party’s apps, e.g., the Apple Health app, to track steps and other physical activity. The exercise tracker is easier to work with smartwatches and other wearable devices for daily use.

#### Food intake

Food tracking typically provides nutritional information (calories, fat, carbohydrates, cholesterol, added sugar content, hidden ingredients, etc.) based on a private food database and/or barcode scanner. This is often useful for customized food to improve blood glucose control. For nonstandardized portion sizes of food, a user can estimate more accurately with “hint” provided by Apps, e.g., using the photo of incremental portion size provided in the Apps as a reference.

#### Medication reminders

Apps in medication reminders are non-diabetic general medication reminders or diabetes-specialised reminders. The former is typically embedded into the common calendar and can be customised by a user. The latter could include recording medications and insulin, calculating insulin doses, and setting up warnings for drug interaction. An advanced feature of medication trackers is to analyse drug responses according to glucose levels.

In Table 3, we provide the features of Apps of Medical (M) and Health & Fitness (HF) categories that are not country-specific and ranked high in at least two countries. Specifically, we removed two Apps, MySweetGestation [68] and myGestationalDiabetes, the former is designed to be an interactive educational tool for both patients and physicians in the field, and the latter’s website does not provide support for our evaluation regarding the features we are interested.

Most diabetes management Apps are good for patients who have been recently diagnosed. The significant advantages of medical diabetes management Apps include: (i) offering a report with more insight into diabetes that can be sent to a healthcare provider to assess the management outcomes, and (ii) setting up an alert system that can automatically send messages to emergency contacts. The diabetes management Apps from the HF category are typically specialised in food, exercise, and lifestyle tracking and recommendation designed for daily use. The built-in trackers in the HF category Apps can be synced in medical diabetes management Apps.

There are many reviews that list top-ranked Apps for diabetes management. In this paper, we aim to overview the proportion of GDM Apps in the diabetes management Apps and provide some insight into whether the diabetes management Apps and devices are sufficient for GDM monitoring. Our evaluation may be limited because we used only one App-ranking search engine, evaluated the IOS Apps only, and searched for the keyword of gestational diabetes only.

## IV Machine Learning Algorithms for GDM Monitoring and Management

Data-driven machine learning algorithms and models are trained to detect patterns and hierarchical casualties in training data and predict future results or make decisions under uncertainty [96]. Statistical and machine learning models are widely used in medical and healthcare data, including generalized linear models (such as logistic regression and linear regression), Bayesian and probability models, deep neural networks, nonparametric models (exemplar-based methods, kernel methods, and trees, forests, bagging and boosting), graph neural networks, generative adversarial networks, transformers, and reinforcement learning. Following the United States Food and Drug Administration (FDA) regulations, artificial intelligence and machine learning-based software are classified as medical devices [97].

In this section, we focus on reviewing machine learning methods and algorithms that have been used in the rapidly expanding field of "Clinical AI" for gestational diabetes and methods that suit a broader family of diabetes (type 1 and type 2 diabetes) that are feasible for GDM monitoring and patient management. These methods are transferable due to (1) the similarity of type 2 diabetes and GDM, and (2) CGM can be used in GDM but is mainly used for patients with type 1 diabetes for timely insulin intervention.

Table 4 summarizes the machine learning models used in gestational studies or models that can potentially be transferred to GDM. There are merely any clinical studies in GDM or type 2 diabetes using CGM. Considering the similarity between GDM and type 2 diabetes, we included models that were developed for hypoglycemia prediction in type 1 and type 2 diabetes studies and the continuous blood glucose prediction using CGM in type 1 diabetes. We will first introduce physiology and hybrid models for measuring and predicting blood glucose levels, and then discuss machine learning models in detail.

### A ML Approaches for Monitoring and Management of Blood Glucose

**Fig. 5:**
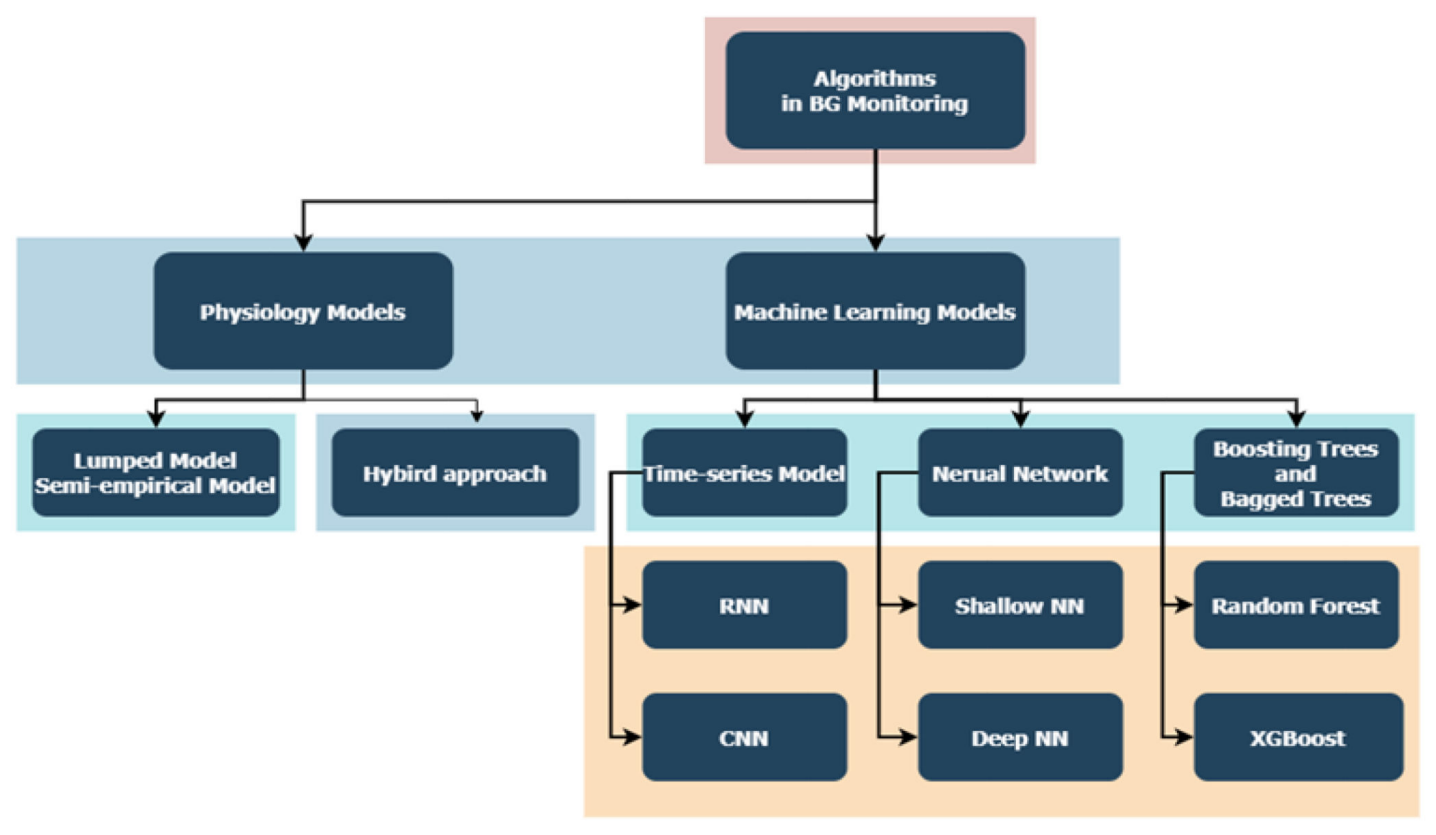
Taxonomy of models for blood glucose prediction.

Machine learning methods used in blood glucose sensor data among different types of diabetes are largely similar, with the mutual aim of facilitating blood glucose monitoring, representing patient electronic health records, and providing timely medication intervention and lifestyle advice to manage diabetes in a personalised and predictive manner. For the prediction of blood glucose, as shown in Fig. 5, there are, in general, two types of prediction models: the physiology-based model and the data-driven machine learning model [98, 99]. As shown in Fig. 5, we used the taxonomy to summarise the model types, and then provided a few exemplary studies with details in this sub-section.

#### a) Physiological Models and Hybrid Method

Physiological models in GDM aim to simulate mother’s and offspring’s glucose-insulin system. There is no physiological model specially developed for mothers with GDM yet, which needs to take offspring’s growth (energy consumption) into consideration. However, it is worth mentioning the need for such models and reviewing existing physiological models that have been developed for general diabetes purposes.

Millsap [100] provided the mathematical analysis of lumped models for the glucose-insulin system, which are made for linear and quadratic inhibition of insulin release. It is a semi-empirical model that the trajectories of the models in the hodograph and time planes are determined, and a comparison of the inhibitory processes is presented.

In the physiological models based on a study by Hovarka [101], the hypothesis is that glucose excursions are influenced by the glucose absorption process, and can be represented as follows:

$$R_{a}\left(t\right)=\frac{CHO_{IN}*CHO_{BIO}*t*e^{\left(-t/t_{max,G}\right)}}{t_{max},G^{2}}$$

Where *t*_max,G_ (min) is the time of the maximum appearance rate of glucose in the accessible glucose compartment, *CHO_IN_* is the number of carbohydrates ingested, and *CHO_BIO_* (dimensionless) is carbohydrate bioavailability. In this model, t was categorised into four periods of 6 h, and was labelled as Nocturnal (01:00 to 06:59 h), *Breakfast* (07:00 to 12:59 h), *Lunch* (13:00 to 18:59 h), and *Dinner* (19:00 h to 00:59).

**Fig. 6:**
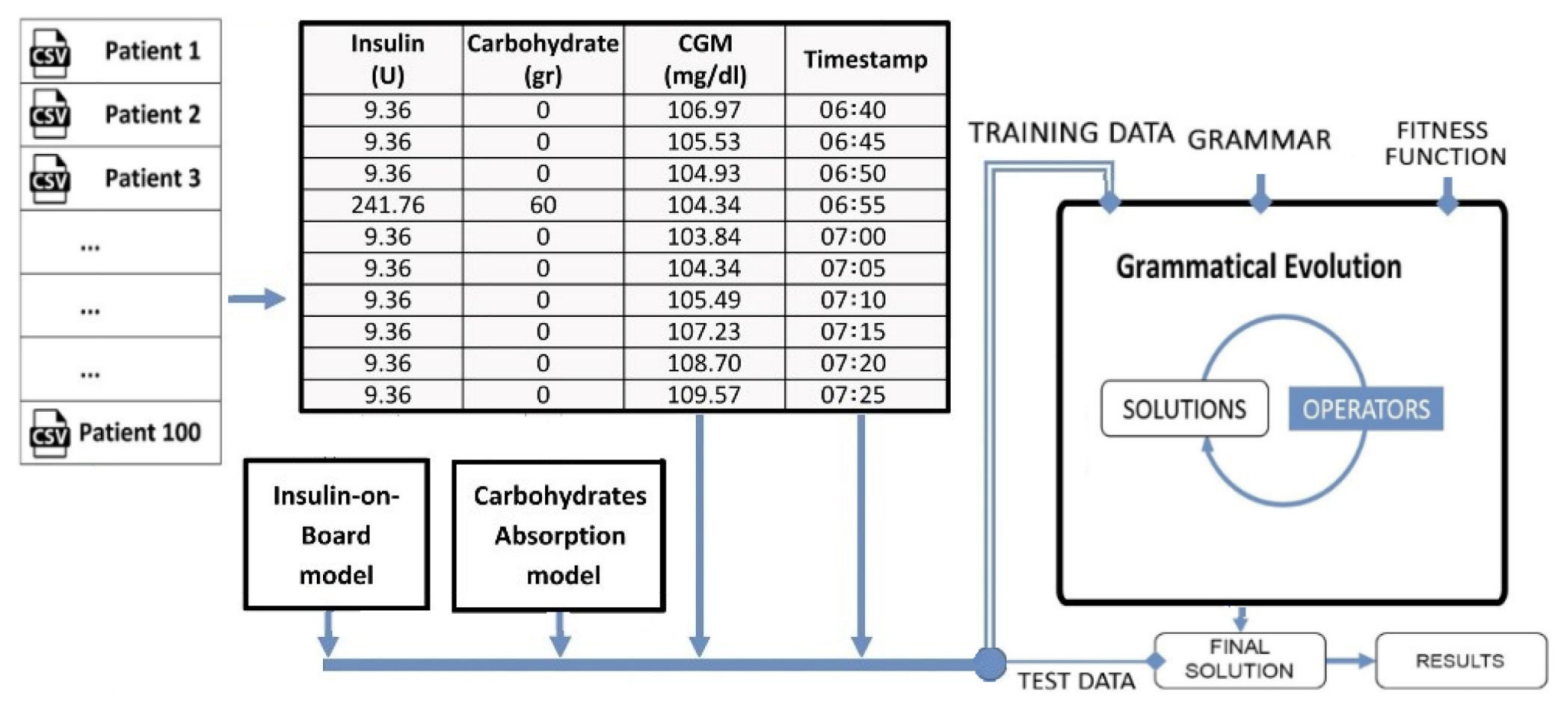
An example of a hybrid approach using the physiological model and data-driven model [76]

Fig. 6 demonstrates a hybrid model that comprises physiological models based on insulin and carbohydrate and a grammatical evolution model. In the data-driven grammatical evolution model [102], the grammar is a population-based heuristic search algorithm that performs an evolutionary process through selection, recombination, and mutation. This method uses a variable-length linear genome to govern how a Backus Naur Form grammar definition is mapped to a program, and expressions and arbitrary complexity programs may evolve. Then the sinusoidal function is added to account for the circadian variations in patients' physiology in the final model with maximum day-to-day 20% amplitude variations.

#### b) Machine Learning Models

##### 1 Blood Glucose Monitoring for GDM

Machine learning technologies for GDM monitoring are premature compared to existing work in type 1 and type 2 diabetes. The main reasons are that the global GDM population is much smaller than the mainstream diabetes population and that type 1 patients widely use CGM compared to GDM patients. Studies that have used ML methods can be divided into those aiming to predict who will develop GDM and those aiming to improve GDM management during or after pregnancies, as listed in Table 4. For mothers with GDM, machine learning models are mainly used to improve blood glucose management, predict clinical outcomes before and after childbirth deliveries, and estimate postnatal risks of type 2 diabetes.

The following studies focused on the prediction and risk evaluation of GDM for early diagnosis.

H. W. Liu et al.’s [69] study used risk scores to predict gestational diabetes in early pregnancy in Tianjin, China. An established population-based prospective cohort of 19,331 pregnant women registered as pregnant before the 15th gestational week. In total, 1484 (7.6%) women developed GDM. The dataset was randomly divided into a training set (70%) and a test set (30%). In this study, the eXtreme gradient boosting (XGBoost) method was employed to predict the presence of GDM. The logistic model was also developed for comparison purposes. Risk factors collected at registration were examined and used to construct the prediction model in the training dataset, including pre-pregnancy body mass index, maternal age, fasting plasma glucose at registration, and alanine aminotransferase. The XGBoost model achieved a higher area under the receiver operating characteristic curve (AUROC) than the logistic model (0.742 vs 0.663, p < 0.001), while the logistic model tended to overestimate the risk at the highest risk level (Hosmer–Lemeshow test p-value: 0.243 vs 0.099).

N. S. Artzi et al. [70] used boosting models for the prediction of GDM based on 588,622 pregnancies in Israel’s nationwide electronic health records. Gradient boosting models predicted GDM with AUROC = 0.85. Results were validated on different geographical validation sets in Israel to emulate real-world performance. Interrogating the boosting models using Sharply value for feature selection, authors developed a risk score table for pre-GDM diagnosis based on nine risk factors.

The Diagnosis of Gestational Diabetes Mellitus (GDM-AI) project [71] implemented an AI model that compared nine algorithms in GDM diagnosis. This is the first prospective and multi-centre clinical study that supports the GDM diagnosis for pregnant women in a resource-restrained setting by using only fasting blood glucose measurement, patient age, and a smartphone connected to the internet. This system was trained on 12,304 pregnant outpatients with their consent, who received a test for GDM in the obstetrics and gynaecology department of the First Affiliated Hospital of Jinan University between November 2010 and October 2017. GDM was diagnosed according to the American Diabetes Association 2011 diagnostic criteria [18]. Age and fasting blood glucose were chosen as critical parameters. Five-fold cross-validation was used for the internal dataset and an external validation dataset that included 1655 cases from the Prince of Wales Hospital, Chinese University of Hong Kong. The AUROC of the external validation dataset for support vector machine (SVM), random forest, AdaBoost, k-nearest neighbours (kNN), Naïve Bayes (NB), decision tree, logistic regression (LR), XGBoost, and gradient boosting decision tree (GBDT) were 0.780, 0.657, 0.736, 0.669, 0.774, 0.614, 0.769, 0.742, and 0.757, respectively. SVM was selected as the method among all nine algorithms. Results showed that the specificity for SVM retained 100% in the external validation set with an accuracy of 88.7%.

Machine learning methods have also been used in finding biomarkers in gestational diabetes. L. Yoffe et al. [72] used a logistic regression model to investigate the role of circulating microRNAs in the plasma of pregnant women in their first trimester. Two populations were included in the study to enable population-specific as well as cross-population inspection of expression profiles. Each microRNA was tested for differential expression in GDM vs control samples. Using both microRNAs in a logistic regression model, the study achieved an AUC value of 0.91. The authors then applied the multivariate models, which achieved an accuracy of mean AUROC = 0.77.

As shown in Table 4, several studies used ML with CGM data in blood glucose prediction for pregnant women with T1 and T2 diabetes. However, ML applications using CGM data in GDM diabetes are limited.

In Pustozerov et al.’s [74] study, linear regression models with lasso regularisation were developed for postprandial glucose response prediction with CGM readings. In this model, the AUC60, AUC120, BG60, Peak BG, the amount and kind of consumed food, the start time of food intake, physical activity, duration of sleep, and the blood glucose were used for model training. Models were evaluated using the correlation coefficient between actual and predicted values (R), root mean square error (RMSE), mean absolute value (MAE) and mean absolute percentage error (MAPE). The prediction results for BG levels 1 hour after food intake were RSME=0.87 mmol/L, MSE=0.69 mmol/L, and MAPE=12.8%, which correspond to an adequate prediction accuracy for BG control decisions. The system was evaluated using the measurement of glucose levels for seven days using the iPro2 CGM with Enlite sensors (Medtronic, Minneapolis, MN, U.S) and independently calibrated with the Accu-Check Performa Nano blood glucose meter (Roche Diabetes Care, Indianapolis, IN, USA) with a minimum of four measurements per day. Linear regression was chosen due to its good interpretability, simplicity, rapid tuning, and adequate accuracy compared to other methods. Two years later, Pustozerov et al.[75] developed another machine learning model using decision tree gradient boosting for postprandial glucose response prediction in women with GDM.

Similar to their study in 2018, this model uses meal-related glycemic index data derived from a mobile App diary, information on previous meals, EHR and patient behavioural questionnaires. This study shows a significant improvement in prediction accuracy compared to their earlier study. Authors reported the best performance model for the prediction of the incremental area under the blood glucose curve two hours after food intake had the following characteristics: R = 0.631, MAE = 0.373 mmol/L*h for the model not using data on current blood glucose; R = 0.644, MAE = 0.371 mmol/L*h for the model using data on the current blood glucose levels; and R = 0.704, MAE = 0.341 mmol/L*h for the model utilizing data on the continuous blood glucose trends before the meal. Based on Shapley additive explanations method, feature ranking results suggested the meal glycemic load, amount of carbohydrates in the meal, type of meal (e.g., breakfast), amount of starch and amount of food consumed 6 hours before the current meal were the most important contributors in the models.

##### 2 Beyond: Continuous Blood Glucose Prediction Methods in Type 1 and Type 2 Diabetes

**Fig. 7:**
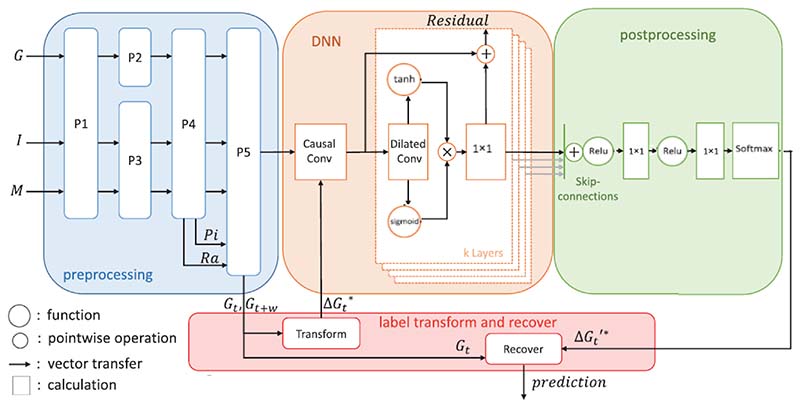
The architecture of GluNet [79].

**Fig. 8:**
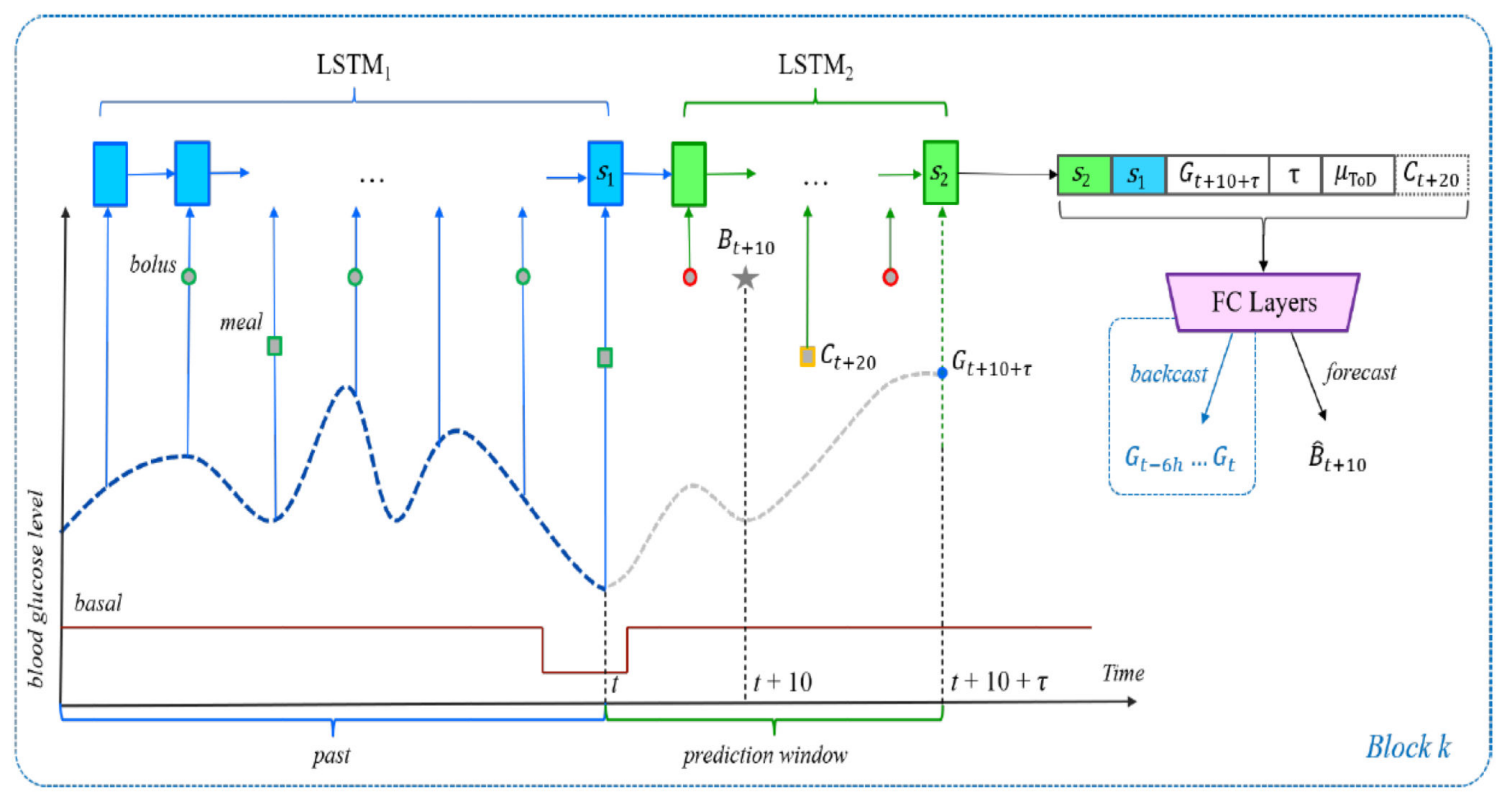
LSTM time-series prediction model with deep residual network [85]

**Fig. 9:**
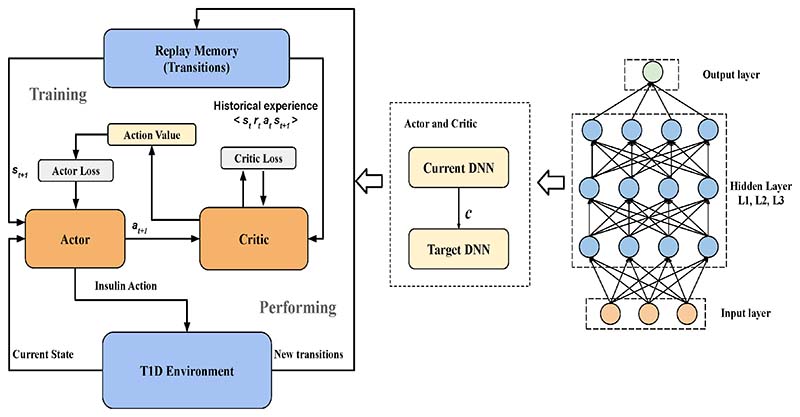
The block diagram of the proposed DRF model with the actor-critic architecture, reproduced without changes from [87]

Thanks to the development of CGM in recent years, there are a significant number of studies in the field of blood glucose prediction using continuous blood glucose measurements, especially for patients with type 1 diabetes. Whilst this paper focuses on machine learning algorithms for GDM management, CGM research to date has mostly focused on type 1 diabetes. Thus we present a review of continuous blood glucose prediction for type 1 and type 2 diabetes that could potentially be adapted for use in GDM patients. Three major machine learning model architectures, including the deep convolutional neural network model (Fig. 7), time-series recurrent neural network (RNN) model (Fig. 8), and reinforcement learning (Fig. 9) architectures are shown to demonstrate machine learning pipelines and their hyperparameters in model designs.

Refiman [77] proposed the autoregressive models to (i) explore the correlations in time-series glucose data and (ii) make blood glucose predictions. Results based on nine type 1 diabetic subjects collected over a continuous 5-day period indicated that, for a 30-minute prediction horizon, individually tuned models yielded 97.6 to 100.0% of data in the clinically acceptable zones A and B. In contrast, cross-subject, portable models yielded 95.8 to 99.7% of data in zones A and B. Due to the small number of patients in this study, the accuracy of the autoregressive model needs to be evaluated in a larger patient cohort.

Mhaskar et al. [81] developed a convolutional neural network (CNN) model to identify the trends of hypoglycemic (0–70 mg/dL), euglycemic (70–180 mg/dL), or hyperglycemic (180–450 mg/dL) based on the 5-min blood glucose prediction. A “judge” network is then used to determine a final prediction based on the outputs of the prediction results for hypoglycemic, euglycemic and hyperglycemic conditions. Methods are evaluated on 25 type 1 diabetes patients’ 160 BG time series data, taken at 5-minute intervals. Diffusion geometry is used to train the networks in a manner analogous to manifold learning. Based on 50% of the training data, this model correctly predicted 96.43% in the hypoglycemic range, 97.96% in the euglycemic range, and 85.29% in the hyperglycemic range.

Zhu [86] used casual dilated CNN layers and WaveNet algorithms to forecast the future glucose levels of patients with type 1 diabetes. The output from the previous layer is the input of the subsequent dilated convolutional layer. The process is repeated until obtaining the final output layer. Then the output is fed into a 1 × 1 convolutional layer followed by the Softmax layer. The model was evaluated on OhioT1DM dataset (6 adolescent subjects) and achieved an RSME of 21.73±2.52 md/dl.

As shown in Fig. 7, Li [79] demonstrates a more complex deep neural network (DNN) architecture called GluNet. This model consists of four parts: pre-processing, DNN, post-processing, and label transformation and recovery. The input data exemplars are CGM time-series measurements *G*, insulin *I* and meal *M,* ; other input factors are optional. As shown in Fig. 7, there are five processes in the preprocessing for data representation: P1 rules out outliers in *G, I, M*; P2 interpolates *G* when the missing data gap is not large; P3 fills or estimates the missing data in *I* and *M*; P4 calculates other factors that should be included as input to the DNN, for instance, plasma insulin estimation *Pi* and glucose rate of appearance *Ra*; and P5 aligns all factors with the same timeline and use them as input to the DNN. The aligned BG time series *Gt* is also sent to the label transform, and quantised *Gt* is used as the category target in training.

Fig. 8 demonstrates an RNN architecture developed by Beauchamp [85] using long short-term memory (LSTM) and a deep residual network for type 1 diabetes management with CGM. The grey star represents the bolus at time t + 10. For the bolus recommendation scenario, the events outlined in red or orange are not allowed in inertial examples. This model was evaluated on the OhioT1DM dataset (12 adolescent subjects) with RSME=13.76.

In addition to supervised and unsupervised machine learning, reinforcement learning is another branch of machine learning methods used for blood glucose prediction. As shown in Fig. 9, Zhu [87] proposed a novel insulin bolus advisor which uses deep reinforcement learning and continuous glucose monitoring to optimize insulin dosing at mealtime. In particular, an actor-critic model based on a deep deterministic policy gradient is designed to compute mealtime insulin doses. The proposed system architecture uses a two-step learning framework, in which a population model is first obtained and then personalized by subject-specific data. Prioritized memory replay is adopted to accelerate the training process in clinical practice. To evaluate the algorithm, an FDA-approved UVA / Padova T1 diabetes simulator was used to perform an in-silico trial on ten adult subjects and ten adolescent subjects. Compared to a standard bolus calculator, the deep reinforcement learning insulin bolus advisor improved the average percentage time in the target range (70–180 mg/dL) from 74.1%±8.4% to 80.9%±6.9% (p<0.01) and 54.9%±12.4% to61.6%±14.1% (p<0.01) in the adult and adolescent cohorts, respectively.

Goldner’s [107] study describes a machine learning method to predict projected blood glucose using 1,923,416 BG measurements from 14,706 people with noninsulin-treated T2 diabetes collected from the One Drop mobile app. Contextual information (CI) on health metrics, including weight and A1c, are included in the demographics. Inputs to each BG prediction included a prior BG and available CI. The model did not distinguish whether BGs with similar CIs were from the same or different users. Forecast horizons were set by the time since the prior BG and varied from 10 minutes to several days. Machine learning methods were not specified in the paper. The median and mean absolute error of holdout predictions were 14.2 and 21.3 mg/dL, respectively, with 91% of predictions within +/-50 mg/dL. Maternal hyperglycemia during pregnancy and delivery is associated with neonatal hypoglycemia and fetal distress. Frequent glucose monitoring is essential to reduce the risk of severe hypoglycemia. Women with well-controlled diabetes and within-range fetal testing may be managed expectantly between 39 and 40 weeks of gestation. However, women with diabetes-related complications, poor glycemic control, or prior stillbirth should be considered for delivery between 36 and 38 weeks of gestation. Readers can find more information on the predictive methods used for hypoglycemia in patients with type 1 diabetes in the review paper [108].

### B Medication and Pregnancy Outcome Management

Due to the individual variability and complex glucose dynamics, optimizing the doses of insulin delivery to minimize the risk of hyperglycemia and hypoglycemia is still a challenge in both CGM and intermittent fingerstick glucose monitoring.

Velardo et al. [94] used machine learning models to identify when a woman with GDM needs to switch to from dietary control to medications (insulin or metformin). Through the analysis of 411,785 blood glucose measurements of 3029 patients, a logistic regression model that can predict the timing of initiation of pharmacological treatment was developed. The authors repeated this experiment on 100 different random permutations of the main dataset between training and validation data using a 70% training and 30% validation split. At each iteration, to avoid biasing the algorithm toward the overrepresented class (diet–diet), this was randomly downsampled to the number of women in the underrepresented class (diet–drug). The lasso function was used with its alpha parameter set to.75 (corresponding to elasticnet regression) and 5-fold cross-validation. After 100 experimental repetitions, they obtained an average area under the receiver operating characteristic curve of 0.80 (SD 0.02) and an algorithm that allows the flexibility of setting the operating point rather than relying on a static heuristic method, which is currently used in clinical practice.

Due to the higher levels of blood glucose in mothers with GDM, offspring will have a higher risk of large-for-gestational-age (LGA) and hyperglycaemia. Using data from a large multi-centre cohort, Gibbons et al [92] created a risk prediction model for LGA infants using logistic regression and naïve Bayes models. Models were developed combining the risks of hyperglycaemia (assessed in three forms: IADPSG GDM yes/no, GDM subtype, OGTT z score quintiles), demographic and clinical variables as potential predictors. Using data from the Hyperglycaemia and Adverse Pregnancy Outcome (HAPO) study [47], authors compared the predictive ability and stability between the models. The two approaches resulted in similar estimates of LGA risk.

In addition to a higher risk of having LGA offspring, excessive gestational weight gain is also associated with poorer pregnancy outcomes [6]. Lu et al. [93] developed machine learning models on 97 patients with GDM to demonstrate a proof-of-the-concept work of caesarean section prediction and to explore the role of temporal blood glucose in predicting caesarean birth. Logistic regression, SVM and Boosting trees were used in model development. The Logistic regression model with Lasso regulator achieved an AUROC of 0.857 ± 0.008. The study also suggested that temporal blood glucose measurements may improve the prediction subject to further validation.

SineDie is a smartphone application with AI that was used during the COVID-19 pandemic [109]. Authors of this paper suggested that it can provide hyperglycaemia prediction and therapy planning, classify and analyse ketonuria, diet transgressions, and blood glucose values, and make recommendations regarding diet or insulin treatment. It automatically prescribed diet therapy modifications, identified the need for insulin treatment and proposed insulin dose changes to doctors. Publication [110] showed that the Expectation Maximization clustering algorithm is first used to group BG measurements in three meal tags: breakfast, lunch, or dinner. Then decision tree is firstly applied to assign each reading into five mealtime tags: "breakfast preprandial","breakfast postprandial", "lunch postprandial","dinner postprandial", or "other", which are then used to classify patients with hyperglycaemia. A randomized clinical trial of 25 GDM patients showed an 88.6% reduction in face-to-face visits and a 27.4% reduction in the time devoted by clinicians to patients’ evaluations. Taking height, weight, and age into account can help advise the patient's initial diet therapy and suggest the total calorie intake distributed in carbohydrate units throughout the day. To calculate the total calorie intake, the authors used the Harris-Benedict equation [111]. This system did not use activity factors or impose any calorie restrictions for obese women. The endocrinologist can modify the personalized diet prescription for each patient. Authors suggested the SineDie system detected all situations requiring therapy adjustment, generating safe recommendations without providing methods used in the decision-making system.

## V Discussion and Future Directions

Digital health and AI technologies offer potential new approaches to improve clinical outcomes and patient experience for women with GDM. By combining digital health techniques and machine learning methods with blood glucose measurements, we can transfer the hard-to-interoperate blood glucose values to actionable insights for intervention [112].

Thanks to the recent developments in GDM management and the need for remote monitoring through the COVID-19 pandemic, several innovations in digital health for GDM are now available to provide patient-centred blood glucose, diet, medication and behavioural management during pregnancy. Whilst there are only a handful of monitoring platforms that have been clinically evaluated, digital platforms are associated with higher satisfaction rates than standard clinical practice. However, the following remain unmet challenges still await to be addressed: (1) scalability and sustainability of known interventions [7]; (2) high cost and other barriers to implementation [16]; (3) engaging women with lifestyle changes during pregnancy [51, 113]; (4) achieving equitable coverage for all women [46]; and (5) using new technologies (e.g., artificial intelligence, smartphones/tablet, telemedicine) in promoting improved glucose monitoring [114, 115].

To resolve these challenges, one question is often asked: can digital health technologies help solve the GDM management crisis? The shortages of clinical resources and the high cost of GDM self-monitoring (either via CGM or daily fingerstick test) created a barrier to cost health inequality. One direction is to consider remote home monitoring (“virtual ward”) and virtual consultation to ease the pressure of shortages in clinical resources. This direction can take advantage of the advances in digital technologies of smartphones, wearables, Apps and machine learning to speed up the adoption of remote monitoring within the national healthcare services. The other direction is to develop risk-based low-cost GDM services that are fit for mothers with different financial and clinical resources. This direction can take advantage of the development of wearable blood glucose sensors and predictive patient monitoring models, which have been reviewed in Sections II and IV of this paper.

From the other perspective, studies in mobile health technologies in blood glucose monitoring have shown the improvement of patient satisfaction in their patient care, but thus far have not shown significant clinical outcome improvement. This is largely due to the lack of clinically-plausible digital health technologies for medication and lifestyle interventions. Meanwhile, large multi-centre or national GDM studies are needed to help identify sub-group of GDM patients thereby improving and redefining the GDM care pathway.

The current key players in the GDM monitoring and management market are hospitals, universities, and companies in the pharmaceutical industry, insurance companies, sensor technology manufacturers and other emerging companies in consumer markets, third-party services and women themselves. These innovators and service providers aim to use digital health technologies to reduce inefficiencies, improve access, reduce costs, increase quality, and make medicine more personalized for patients. Early identification, referral and management of pregnant women at increased risk may offer opportunities for prevention. Innovations for pre-GDM and post-pregnancy health monitoring are also urgently needed; these innovations can enable individuals and healthcare providers to estimate the risk of gestational diabetes and type 2 diabetes and provide timely intervention.

There are other challenges limiting the digital health innovations for GDM monitoring. Firstly, despite the advances in AI-enabled technologies, there remain issues in model interpretability, trustworthiness, fairness and ethics for end-users and service providers. Beyond the technical and technology challenges, evidence-based and quantitative analysis of improvement in patient health and economic costs need to be evaluated in epidemiology studies for specified populations (community, city, state, country, global, or specific to patients with certain diseases). Secondly, the accuracy and cost of sensor technologies in glucose monitoring have yet to be improved. CGM fills a gap that exists in diabetes monitoring and treatment. It provides continuous readings and thereby can continuously analyze and respond to an individual's glucose levels. Another advantage of CGM comes from its nature as “wearable”. Nevertheless, patients need to change their sensors every three to seven days, making it an effective but expensive solution that many people and health systems cannot afford. There is still a lack of applications in monitoring blood glucose that can be fused into finger-tip periodic blood glucose sensors.

In the following, we suggest some promising directions for future research: (i) early diagnosis of GDM: proven genetic and/or placental extracellular vesicle bound biomarkers should be taken into consideration for the early diagnosis of GDM [6, 116]. The pre-diabetes population will benefit from this improvement [117]; (ii) Management of gestational diabetes during pregnancy: This should be personalised on the basis of underlying pathogenesis and response to different management strategies. Future GDM screening strategies should include specific treatment guidelines for patients with companion diseases, such as obesity and cardiovascular disease. Therefore, patients at high risk of complications can be informed and treated in a timely manner: machine learning is an ideal tool for this development; (iii) Postnatal type 2 diabetes and cardiovascular disease prevention: national level postnatal screening programmes, large-scale clinical research and digital health applications for postnatal monitoring are urgently needed to delay the development of type 2 diabetes and cardiovascular disease. (iv) Health economic research: global prevalence of GDM varies; the rate of GDM was shown doubled in pregnant South East Asian women than in Caucasian women [118]. National and local health economic analyses are needed for developing country-specific cost-effectiveness models that incorporate the cost of any new technologies, alongside treatment costs and long-term healthcare costs for the health system, as well as directly for the mother and child. This will assist policymakers and service providers in prioritising whether novel technologies make financial sense in different care systems around the world. (v) Low-cost blood glucose monitoring solutions are urgently needed for low-and-middle-income countries. (vi) Development of other potential wearable techniques include optical-based blood glucose estimation methods on wearable or portable devices and wearable contact lenses for blood glucose monitoring. (vii) Expanding the physiology knowledge map in GDM to understand and quantitatively address the association among diabetes, pregnancy, and human metabolism, as well as other health conditions, such as hypertension, sleep disorder, postnatal depression, and cardiovascular diseases.

In conclusion, AI and ML are promising, emerging areas for the monitoring and management of women with gestational diabetes. While ML and AI have proven to be useful in research and clinical practices for patient monitoring using risk stratification, patient subgroup discovery, and natural language processing-based outcome prediction models [119–130], similar approaches for GDM are yet to be developed. The exciting field of collecting data from different sensors for activity tracking, food intake quantification, blood glucose monitoring and medication management could open up new possibilities to help people manage GDM. However, there are still many unanswered questions for data scientists, engineers and clinicians. Personalized, explainable, and trustful AI and ML models are needed to assist patients and clinicians in improving patients’ lifestyles and short-term and long-term clinical outcomes. There is an urgent need to develop digital health technologies and explainable AI methods to identify patients at different risk groups at an earlier stage (preventive medicine) and provide clinicians with a reactive treatment plan using predictive monitoring models.

## Figures and Tables

**Table 1:** Patient monitoring technologies in GDM clinical studies: primary purpose, outcome measures, monitoring methods, country of use, and reviewer comments

Application/First Authors (Year) [reference]	Study title	Location, Phase (study size)	Outcome Measures	Study type: Primary purpose
**Treat-GDM** (2013-2016) [52, 53]	Trial of Remote Evaluation and Treatment of Gestational Diabetes Mellitus	UK, N/A (203)	Glycosylated haemoglobin; mean Blood glucose levels for fasting, preprandial and postprandial readings; percentage of ‘on target’ blood glucose readings; effectiveness of monitoring; maternal outcomes; maternal weight gain; birthweight; birth injury; neonatal hypoglycaemia.	Behavioural: Health services research, self home blood glucose monitoring
**PEARS** (2013-2016) [62]	Pregnancy, Exercise And nutrition Research study with app support	Ireland, N/A (750)	Effectiveness of a smartphone-assisted targeted healthy lifestyle intervention; support antenatal management of overweight and obese pregnant population in preventing GDM.	Interventional
**Hola Bebe** (2014-2017)	Mobile Health App to Reduce Diabetes in Latina Women	USA, Phase 1 (18)	Self-efficacy for healthy eating; physical activity; body weight.	Behavioural: Prevention
**MobiGuide** (2014) [55, 56]	Group and Mobile Care for Gestational Diabetes	Spain, N/A (22)	Patient data automatically or manually entered in App; decision support system generates feedback to patients and clinicians based on clinical guidelines.	Interventional
**Pregnant**+ (2015-2017) [58]	A Mobile Smartphone Application to Promote a Healthy Diet and Physical Activity Among Pregnant Women With GDM	Norway, N/A (238)	OGGT; dietary intake; motivation for eating healthy; physical activity; motivation for physical activity; depression; complications of pregnancy; mode of delivery and complications at birth for the mother; complications for the newborn.	Interventional: Prevention
**Pregnant**+ (2019-2022)	Effects of Dietary and Weight Management on Pregnancy Outcomes in Mobile Medical Platform	China, N/A (2000)	Incidence of gestational diabetes mellitus, gestational hypertension, caesarean section and premature birth.	Behavioural: Intensive dietary; weight management; standard care
**Pregnant**+ (2021-2022)	Development and Testing of a Mobile Health Application for Management of Gestational Diabetes	Nepal, N/A (60)	Maternal blood glucose levels at 6 weeks postpartum; neonatal birth weight; induction of labour; caesarean delivery; self-monitoring adherence; usability of telemonitoring; app acceptability and usability.	Behavioural: Supportive care.
**University of Colorado Hospital** (2015-2021)	User Testing and Feedback for a Mobile Health Program for Postpartum Women: A Pilot Study	USA, N/A (200)	Usability of the application; engagement with the application; navigability of the application; acceptability of the application	Observational
**Peking Union Medical College Hospital** (2016-2020)	the Effect of Mobile Medical Used for the Standardized Management of Gestational Diabetes	China, N/A (400)	Glycaemic qualification rate; pregnancy outcome.	Behavioural: Prevention, intensive dietary, weight management and standard care
**iHealth** (2017-2018)	Evaluating the Feasibility of Using M-Health to Improve Serum Glucose Logs	USA, N/A (8)	Glucose log completeness; patient satisfaction.	Interventional
**FAB** (2017-2020)	Fit After Baby: Increasing Postpartum Weight Loss in Women at Increased Risk for Cardiometabolic Disease	USA, N/A (34)	Changes in weight loss, postpartum weight retention, waist circumference fasting insulin, fasting glucose HbA1c; adherence to self-monitoring; Satisfaction; use of App; number of interactions with lifestyle coaches	Interventional: Prevention
**LIVING** (2017-2021)	Lifestyle Intervention IN Gestational Diabetes	Bangladesh and India, N/A (1612)	Change of glycemic category; changes in fasting blood glucose; change in body weight; change in waist circumference; change in systolic blood pressure; change in physical activity level; change in dietary habits.	Interventional: Prevention
**SweetMama** (2017-2021) [104, 105]	SweetMama: Testing of a Novel Technology for Diabetes Education and Support to Pregnant Women	USA, N/A (80)	Focus group and individual user feedback; feasibility; user interactivity data; usability testing; diabetes self-efficacy; patient activation; difference in HbA1C	Interventional: Supportive care
**GlucoseMama** (2018-2019)	Group and Mobile Care for Gestational Diabetes	USA, N/A (22)	Reduction in the number of pharmacotherapy for treatment, neonates born large, and infants with neonatal hypoglycemia; increased number of screened in the postpartum period for type 2 diabetes.	Interventional: supportive care
**eMoMs** (2019-2022)	Usability Study of the Sensors and eMoM GDM Application	Finland, N/A (34)	Acceptability and usability of sensors; acceptability of prototype app; technical functionality.	Observational
**MELINDA** (2019-2022) [63]	Mobile-based Lifestyle Intervention in Women With Glucose Intolerance After Gestational Diabetes	Belgium, N/A (236)	Metabolic syndrome; insulin resistance Matsuda; insulin resistance HOMA-IR beta-cell function ISSI-2 index; beta-cell function insulinogenic index; beta-cell function HOMA-B; weight loss	Behavioural: Prevention
**GUIDES** (2021-2022)	Gestational Diabetes in Uganda and India Improving Screening and Self-management	India; Uganda, N/A (20000)	GDM diagnosis; mean fasting blood glucose; adverse perinatal outcomes (composite measure); HbAlc	Interventional
**SMARThealth** (2019-2020) [103]	SMARThealth Pregnancy: Feasibility & Acceptability of a Complex Intervention for High-risk Pregnant Women in Rural India	India, N/A (258)	Recruitment rate; retention rate; number of home visits; number of women with GDM; postpartum follow-up; number of hypertensive disorders; number of severe anaemia; mean postpartum haemoglobin; mean postpartum; Systolic and diastolic blood pressure.	Interventional: Prevention
**Johns Hopkins Health System and University** (2021-2024)	Pragmatic Randomized Clinical Trial to Limit Weight Gain in Pregnancy and Prevent Obesity	USA, N/A (380)	Total gestational weight gain; number of participants who gained excess weight; incidence of GDM; postpartum weight retention; infant weight; proportion of low birth weight infants	Interventional: Health services research

**Table 2:** Top 10 IOS apps ranked seven countries with high GDM prevalence

Countries	Medical (M)	Health & Fitness (HF)	Food & Drink (FD)	lifestyle (LS)
**SGP** *	Glucose tracker ++ MySweetGestation gluQUO: Control your Diabetes Pregnant with diabetes	myGestationalDiabetes My Diabetes Diet & Meal Plan Klinio: Diabetic Diet Log Habits: Gestational Diabetes Cori – Better Diabetes		Melinda
**UAE** *	FreeStyle LibreLink – AE Alma Health Glucose Buddy Diabetes Tracker Medisage Phill Reminder MySweetGestation	myGestationalDiabetes Habits: Gestational Diabetes Carb Manager: Keto Diat App Calorie Counting App Withings Health Mate		
**THAI** *	DMThai Diary Medisafe Phill Reminder Glucose Buddy Diabetes Tracker mySugr- Diabetes Tracker Log	Habits: Gestational Diabetes myGestationalDiabetes Life Fasting Progress Tracker Withings Health Mate One Drop Diabetes Management	My Diabetic Meal Planner	
**MAL** *	Glucose Buddy Diabetes Tracker Medisafe Phill Reminder Glucose – Blood Sugar racker Diabetes: M Pregnant with Diabetes	myGestationalDiabetes Habits: Gestational Diabetes Carb Manager: Keto Diet App Life Fasting Progress Tracker One Drop Diabetes Management		
**UK** *	GDm-Health mySugr- Diabetes Tracker Log Glucose tracker ++ Hedia -Personal Diabetes App	myGestationalDiabetes Diabetes Diary Mumoactive Diabetes	Diabetes Recipe App Diabetes Diet FREE – Proper Nutrition for the Diabetic	
**BEL** *	One Touch Reveal FreeStyle LibreLink – BE mySugr-DiabetesTracker LogMySweetGestation	Habits: Gestational Diabetes myGestationalDiabetes Withings Health Mate Pacer Pedometer & Step Tracker Carb Manager: Keto Diat App		
**IND** *	mySugr- Diabetes Tracker Log One Touch Reveal Glucose Buddy Diabetes Tracker Medisafe Phill Reminder Glucose – Blood Sugar racker	Habits: Gestational Diabetes myGestationalDiabetes BeatO Biabetes Management Withings Health Mate One Drop Diabetes Management		

* SGP: Singapore, UAE: United Arab Emirates, THAI: Thailand, MAL: Malaysia, UK: United Kingdom, BEL: Belgium, IND: India

**Table 3:** Features of different diabetes management apps

App Name and Type	Glucose Monitoring with CGM	Exercise Response	Food Tracking	Medication Reminders	Interaction with Doctors
**mySugr- Diabetes Tracker Log (M)** [67]	✓		✓	✓	
**Diabetes: M (M)**	✓		✓	✓	✓
**Glucose Buddy Diabetes Tracker (M)**	✓		✓		
**Medisage Phill Reminder (M)**				✓	✓
**Habits: Gestational Diabetes (HF)**	✓	✓	✓	✓	
**Carb Manager: Keto Diet App (HF)**		✓	✓		
**Withings Health Mate (HF)**		✓	✓		
**One Drop Diabetes Management (HF)**	✓	✓	✓	✓	

**Table 4:** Machine learning models used in gestational diabetes and type 1 and type 2 diabetes studies

Aim	Model	Previously Used For:			Publication
		Type 1 *	Type 2 *	GDM	
**Early Diagnosis of GDM including biomarker detection**	Boosting Models			✓	[69–71]
	Random Forest			✓	[71]
	Generalized Linear Model			✓	[71, 72, 84]
	K-Nearest Neighbour			✓	[71]
	Bayesian, include Naïve Bayes			✓	[71]
	Support Vector Machine			✓	[71]
**Hypoglycemia Prediction**	Random forest		✓		[73]
	Support Vector Machine		✓		[73]
	k-nearest neighbour		✓		[73]
	Bayesian, include Naïve Bayes		✓		[73]
**Continuous Blood Glucose Prediction**	Linear regression with Lasso			✓	[74]
	Boosting Model			✓	[75]
	Physiological models	✓			[76]
	Autoregressive Model	✓			[77–79]
	Bayesian, include Naïve Bayes		✓		[80]
	Deep Neural Network	✓	✓		[79, 81, 83]
	Convolutional Neural Network	✓			[78, 79, 85, 86]
	Reinforcement Learning	✓			[87]
	Long Short Term Memory	✓			[78, 85, 88]
	Support Vector Regressor	✓			[79]
	Transfer Learning	✓	✓		[89–91]
**Pregnancy Outcome Prediction**	Logistic Regression			✓	[92, 93]
	Bayesian, include Naïve Bayes			✓	[92]
	Boosting Models			✓	[93]
	Tree-based Models			✓	[93]
	Support Vector Machine			✓	[93]
**Medication and Clinical Review Management**	Logistic Regression			✓	[82, 94, 95]
	Random Forest			✓	[82]
	Boosting Models			✓	[82, 95]
**Postnatal Type 2 Diabetes Prediction**	Logistic Regression			✓	[106]
	Support Vector Machine			✓	[106]
	Boosting Models			✓	[106]
	Neural Network			✓	[106]

* Type 1 and type 2 applications were only used when there were limited applications in gestational diabetes

## References

[1] Alberti KG, Zimmet PZ (1998). Definition, diagnosis and classification of diabetes mellitus and its complications. Part 1: diagnosis and classification of diabetes mellitus provisional report of a WHO consultation. Diabet Med.

[2] Garrison A (2015). Screening, diagnosis, and management of gestational diabetes mellitus. Am Fam Physician.

[3] Zhu WW (2017). High prevalence of gestational diabetes mellitus in Beijing: effect of maternal birth weight and other risk factors. Chin Med J.

[4] Martis R CC (2018). Treatments for women with gestational diabetes mellitus: an overview of Cochrane systematic reviews. Cochrane Db Syst Rev.

[5] International Diabetes Federation "IDF diabetes altas".

[6] Saravanan P (2020). Gestational diabetes: opportunities for improving maternal and child health. Lancet Diabetes Endocrinol.

[7] Crowther CA (2005). Effect of treatment of gestational diabetes mellitus on pregnancy outcomes. N Engl J Med.

[8] Landon MB (2009). A multicenter, randomized trial of treatment for mild gestational diabetes. New Engl J Med.

[9] Bellamy L (2009). Type 2 diabetes mellitus after gestational diabetes: a systematic review and meta-analysis. Lancet.

[10] Kim C (2014). Maternal outcomes and follow-up after gestational diabetes mellitus. Diabet Med.

[11] Catalano PM, Drago NM, Amini SB (1998). Longitudinal changes in pancreatic beta-cell function and metabolic clearance rate of insulin in pregnant women with normal and abnormal glucose tolerance. Diabetes Care.

[12] Organization WH (2014). Diagnostic criteria and classification of hyperglycaemia first detected in pregnancy: a World Health Organization Guideline. Diabetes Res Clin Pract.

[13] NICE (2020). Diabetes in pregnancy: management from preconception to the postnatal period.

[14] NICE (2018). Diabetes in pregnancy overview.

[15] NICE (2018). Gestational Diabetes: Risk assessment, testing, diagnosis and management.

[16] Mackillop L (2018). Comparing the efficacy of a mobile phone-based blood glucose management system with standard clinic care in women with gestational diabetes: Randomized controlled trial. JMIR Mhealth Uhealth.

[17] Bhatia M (2018). Clinical implications of the NICE 2015 criteria for gestational diabetes mellitus. J Clin Med.

[18] Association AD (2011). Standards of medical care in diabetes—2011. Diabetes Care.

[19] (2020). What is digital health.

[20] Loerup L (2014). GDm-Health: a pilot study examining acceptability of mobile phone assisted remote blood glucose monitoring for women with gestational diabetes. Diabetic Med.

[21] GDm-Health app wins innovation award.

[22] Blood glucose monitoring.

[23] Clarke SE, Foster JR (2012). A history of blood glucose meters and their role in self-monitoring of diabetes mellitus. Brit J Biomed Sci.

[24] Burrin JM, Price CP (1985). Measurement of blood glucose. Annals of Clinical Biochemistry.

[25] Benjamin EM (2002). Self-monitoring of blood glucose: The basics. Clinical Diabetes.

[26] Olansky L, Kennedy L (2010). Finger-stick glucose monitoring issues of accuracy and specificity. Diabetes Care.

[27] Self-monitoring blood glucose test systems for over-the-counter use: Guidance for industry and good and drug administration staff.

[28] Klonoff DC (2018). Investigation of the accuracy of 18 marketed blood glucose monitors. Diabetes Care.

[29] Klonoff DC (2005). Continuous glucose monitoring roadmap for 21st century diabetes therapy. Diabetes Care.

[30] Didyuk O Continuous glucose monitoring devices: Past, present, and future focus on the history and evolution of technological innovation. Journal of Diabetes Science and Technology.

[31] Klonoff DC (2005). Continuous glucose monitoring: Roadmap for 21st century diabetes therapy. Diabetes Care.

[32] Thulasi AA (2017). Portable impedance measurement device for wweat based glucose detection. Int Conf Wearab Impl.

[33] Lee H (2018). Enzyme-based glucose sensor: From invasive to wearable device. Adv Healthc Mater.

[34] Kim J, Campbell AS, Wang J (2018). Wearable non-invasive epidermal glucose sensors: A review. Talanta.

[35] Huang JM, Zhang Y, Wu J (2020). Review of non-invasive continuous glucose monitoring based on impedance spectroscopy. Sensor Actuat a-Phys.

[36] van Enter BJ, von Hauff E (2018). Challenges and perspectives in continuous glucose monitoring. Chem Commun.

[37] Siddiqui SA (2018). Pain-free blood glucose monitoring using wearable sensors: Recent advancements and future prospects. IEEE reviews in biomedical engineering. Research Support, Non-U.S. Gov’t; Review.

[38] Jernelv IL (2019). A review of optical methods for continuous glucose monitoring. Appl Spectrosc Rev, Review.

[39] Ellis DI, Goodacre R (2006). Metabolic fingerprinting in disease diagnosis: biomedical applications of infrared and Raman spectroscopy. Analyst.

[40] Hanna J (2020). Noninvasive, wearable, and tunable electromagnetic multisensing system for continuous glucose monitoring, mimicking vasculature anatomy. Science Advances.

[41] Chen YH (2017). Skin-like biosensor system via electrochemical channels for noninvasive blood glucose monitoring. Science Advances.

[42] Rachim VP, Chung WY (2019). Wearable-band type visible-near infrared optical biosensor for non-invasive blood glucose monitoring. Sensor Actuat B-Chem.

[43] Chen Q, Carbone ET (2017). Functionality, implementation, impact, and the role of health literacy in mobile phone Apps for gestational diabetes: Scoping review. JMIR Diabetes.

[44] Raman P (2017). Different methods and settings for glucose monitoring for gestational diabetes during pregnancy. Cochrane Db Syst Rev.

[45] Egan AM, Dow ML, Vella A (2020). A review of the pathophysiology and management of diabetes in pregnancy. Mayo Clinic Proceedings.

[46] Wendland EM (2012). Gestational diabetes and pregnancy outcomes-a systematic review of the World Health Organization (WHO) and the International Association of Diabetes in Pregnancy Study Groups (IADPSG) diagnostic criteria. BMC Pregnancy Childbirth.

[47] Freathy RM (2010). Hyperglycemia and adverse pregnancy outcome (HAPO) study. Diabetes.

[48] Nguyen M (2021). Systematic evaluation of Canadian diabetes smartphone applications for people with type 1, type 2 and gestational diabetes. Can J Diabetes.

[49] Ding GF (2012). Evaluation of continuous glucose monitoring (CGM) on gestational diabetes mellitus in China. Diabetes.

[50] NICE Health App: GDm-Health for people with gestational diabetes.

[51] Hirst JE (2015). Preventing childhood obesity starts during pregnancy. Lancet.

[52] Hirst JE (2015). GDm-health: development of a real-time smartphone solution for the management of women with gestational diabetes mellitus (GDM). Bjog-Int J Obstet Gy.

[53] Loerup L (2015). A comparison of blood glucose metrics to assess the feasibility of a digital health system for management of women with gestational diabetes: the GDm-Health study. Diabetic Med.

[54] Dyson PA (2018). GDm-Health Plus: Development of a remote behavioural lifestyle management system for women with gestational diabetes. Diabetic Med.

[55] Peleg M (2017). MobiGuide: a personalized and patient-centric decision-support system and its evaluation in the atrial fibrillation and gestational diabetes domains. User Model User-Adap.

[56] García-Sáez G (2014). Patient-oriented computerized clinical guidelines for mobile decision support in gestational diabetes. Journal of Diabetes Science and Technology.

[57] Garnweidner-Holme LM (2015). Designing and developing a mobile smartphone application for women with gestational diabetes mellitus followed-up at diabetes outpatient clinics in Norway. Healthcare-Basel.

[58] Borgen I (2017). Smartphone application for women with gestational diabetes mellitus: a study protocol for a multicentre randomised controlled trial. Bmj Open.

[59] Borgen I (2019). Effect of the pregnant plus smartphone application in women with gestational diabetes mellitus: a randomised controlled trial in Norway. Bmj Open.

[60] Skar JB (2018). Women's experiences with using a smartphone app (the Pregnant plus app) to manage gestational diabetes mellitus in a randomised controlled trial. Midwifery.

[61] Ainscough KM (2019). Nutrition, behavior change and physical activity outcomes from the PEARS RCT-An mHealth-supported, lifestyle intervention among pregnant women with overweight and obesity. Front Endocrinol).

[62] Kennelly MA (2016). Pregnancy, exercise and nutrition research study with smart phone app support (Pears): Study protocol of a randomized controlled trial. Contemp Clin Trials.

[63] Minschart C (2020). Mobile-based lifestyle intervention in women with glucose intolerance after gestational diabetes mellitus (MELINDA), a multicenter randomized controlled trial: Methodology and design. Journal of Clinical Medicine.

[64] Jacobson LT (2020). Electronic monitoring of mom's schedule (eMOMS™): Protocol for a feasibility randomized controlled trial to improve postpartum weight, blood sugars, and breastfeeding among high BMI women. Contemporary Clinical Trials Communications.

[65] Chen R (2003). Continuous glucose monitoring for the evaluation and improved control of gestational diabetes mellitus. J Matern Fetal Neonatal Med.

[66] Zhu Y, Zhang C (2016). Prevalence of gestational diabetes and risk of progression to type 2 diabetes: A global perspective. Curr Diab Rep.

[67] Debong F, Mayer H, Kober J (2019). Real-World Assessments of mySugr Mobile Health App. Diabetes Technol Ther.

[68] Tumminia A (2019). “MySweetGestation”: A novel smartphone application for women with or at risk of diabetes during pregnancy. Diabetes Res Clin Pract.

[69] Liu HW (2021). Machine learning risk score for prediction of gestational diabetes in early pregnancy in Tianjin, China. Diabetes-Metab Res.

[70] Artzi NS (2020). Prediction of gestational diabetes based on nationwide electronic health records. Nature Medicine.

[71] Shen JY (2020). An innovative artificial intelligence-based App for the diagnosis of gestational diabetes mellitus (GDM-AI): development study. Journal of Medical Internet Research.

[72] Yoffe L (2019). Early diagnosis of gestational diabetes mellitus using circulating microRNAs. Eur J Endocrinol.

[73] Sudharsan B, Peeples MM, Shomali ME (2015). Hypoglycemia prediction using machine learning models for patients with type 2 diabetes. Journal of Diabetes Science and Technology.

[74] Pustozerov E (2018). Development and evaluation of a mobile personalized blood glucose prediction system for patients with gestational diabetes mellitus. JMIR mHealth and uHealth.

[75] Pustozerov EA (2020). Machine learning approach for postprandial blood glucose prediction in gestational diabetes mellitus. Ieee Access.

[76] Contreras I (2017). Personalized blood glucose prediction: A hybrid approach using grammatical evolution and physiological models. PLoS ONE.

[77] Reifman J (2007). Predictive Monitoring for Improved Management of Glucose Levels. Journal of Diabetes Science and Technology.

[78] Xie J, Wang Q (2020). Benchmarking machine learning algorithms on blood glucose prediction for type 1 diabetes in comparison with classical time-series models. IEEE Transactions on Biomedical Engineering.

[79] Li K (2020). GluNet: A deep learning framework for accurate glucose forecasting. IEEE Journal of Biomedical and Health Informatics.

[80] Mueller L (2020). Application of machine learning models to evaluate hypoglycemia risk in type 2 diabetes. Diabetes Therapy.

[81] Mhaskar HN, Pereverzyev SV, Van Der Walt MD (2017). A deep learning approach to diabetic blood glucose prediction. Frontiers in Applied Mathematics and Statistics.

[82] Liao LD (2022). Development and validation of prediction models for gestational diabetes treatment modality using supervised machine learning: a population-based cohort study. BMC Med.

[83] Faruqui SHA (2019). Development of a deep learning model for dynamic forecasting of blood glucose level for type 2 diabetes mellitus: Secondary analysis of a randomized controlled trial. JMIR mHealth and uHealth.

[84] Muche AA, Olayemi OO, Gete YK (2020). Predictors of postpartum glucose intolerance in women with gestational diabetes mellitus: a prospective cohort study in Ethiopia based on the updated diagnostic criteria. BMJ Open.

[85] Beauchamp J (2021). LSTMs and deep residual networks for carbohydrate and bolus recommendations in type 1 diabetes management. Sensors.

[86] Li K (2020). Convolutional recurrent neural networks for glucose prediction. IEEE Journal of Biomedical and Health Informatics.

[87] Zhu T (2020). An insulin bolus advisor for rype 1 diabetes using deep reinforcement learning. Sensors.

[88] Aliberti A (2019). A multi-patient data-driven approach to blood glucose prediction. IEEE Access.

[89] Deng Y (2021). Deep transfer learning and data augmentation improve glucose levels prediction in type 2 diabetes patients. NPJ Digital Medicine.

[90] De Bois M, El Yacoubi MA, Ammi M (2021). Adversarial multi-source transfer learning in healthcare: Application to glucose prediction for diabetic people. Computer Methods and Programs in Biomedicine.

[91] Gupta P (2020). Transfer learning for clinical time series analysis using deep neural networks. Journal of Healthcare Informatics Research.

[92] Gibbons KS (2021). Prediction of large-for-gestational age infants in relation to hyperglycemia in pregnancy – A comparison of statistical models. Diabetes Res Clin Pr.

[93] Lu H (2022). Standardising the assessment of caesarean birth using an oxford caesarean prediction score for mothers with gestational diabetes. Healthcare Technology Letters.

[94] Velardo C (2021). Toward a Multivariate Prediction Model of Pharmacological Treatment for Women With Gestational Diabetes Mellitus: Algorithm Development and Validation. J Med Internet Res.

[95] Yang J (2022). Machine Learning-Based Risk Stratification for Gestational Diabetes Management. Sensors (Basel).

[96] Murphy KP (2012). Machine learning: A probabilistic perspective.

[97] (2021). Artificial Intelligence and Machine Learning in Software as a Medical Device.

[98] Oviedo S (2017). A review of personalized blood glucose prediction strategies for T1DM patients. International Journal for Numerical Methods in Biomedical Engineering.

[99] Woldaregay AZ (2019). Data-driven blood glucose pattern classification and anomalies detection: machine-learning applications in rype 1 diabetes. Journal of Medical Internet Research.

[100] Millsaps K, Pohlhausen K (1975). A mathematical model for glucose-insulin interaction. Mathematical Biosciences.

[101] Hovorka R (2004). Nonlinear model predictive control of glucose concentration in subjects with type 1 diabetes. Physiol Meas.

[102] Ryan C, Collins J, Neill MO (1998). Grammatical evolution: Evolving programs for an arbitrary language. Springer Berlin Heidelberg.

[103] Nagraj S (2021). SMARThealth pregnancy: Feasibility and ccceptability of a complex intervention for high-risk pregnant women in rural India: Protocol for a pilot cluster randomised controlled trial. Front Glob Womens Health.

[104] Yee LM (2020). SweetMama: Usability testing of a novel mobile application for diabetes education and support during pregnancy. Am J Obstet Gynecol.

[105] Yee LM (2021). Patient and provider perspectives on a novel mobile health intervention for low-income pregnant women with gestational or type 2 diabetes mellitus. J Diabetes Sci Technol.

[106] Kumar M (2022). Machine learning–derived prenatal predictive risk model to guide intervention and prevent the progression of gestational diabetes mellitus to type 2 diabetes: Prediction model development study. JMIR Diabetes.

[107] Goldner DR (2018). A machine-learning model accurately predicts projected blood glucose. Diabetes.

[108] Mujahid O, Contreras I, Vehi J (2021). Machine learning techniques for hypoglycemia prediction: Trends and challenges. Sensors.

[109] Albert L (2020). Managing gestational diabetes mellitus using a smartphone application with artificial intelligence (SineDie) during the COVID-19 pandemic: Much more than just telemedicine. Diabetes Res Clin Pr.

[110] Caballero-Ruiz E (2017). A web-based clinical decision support system for gestational diabetes: Automatic diet prescription and detection of insulin needs. International Journal of Medical Informatics.

[111] Harris JA, Benedict FG (1918). A biometric study of human basal metabolism. Proc Natl Acad Sci U S A.

[112] Reiterer F (2017). Significance and reliability of MARD for the accuracy of CGM systems. J Diabetes Sci Technol.

[113] Maitland RA (2014). Prediction of gestational diabetes in obese pregnant women from the UK pregnancies better eating and activity (UPBEAT) pilot trial. Diabet Med.

[114] Walker E, Flannery O, Mackillop L (2020). Gestational diabetes and progression to type two diabetes mellitus: missed opportunities of follow up and prevention?. Prim Care Diabetes.

[115] Hodgkinson JA (2014). Is self monitoring of blood pressure in pregnancy safe and effective?. Bmj-Brit Med J.

[116] Kandzija N (2019). Placental extracellular vesicles express active dipeptidyl peptidase IV; levels are increased in gestational diabetes mellitus. J Extracell Vesicles.

[117] Cappon G (2019). Continuous glucose monitoring sensors for diabetes management: A review of technologies and applications. Diabetes Metab J.

[118] Li R (2022). Graph signal processing, Graph neural network and graph learning on biological data: A systematic review. IEEE Rev Biomed Eng.

[119] Zhou B, Yang G, Shi Z, Ma S (2022). Natural language processing for smart healthcare. IEEE Rev Biomed Eng.

[120] Giuste F (2022). Explainable artificial intelligence methods in combating pandemics: A systematic review. IEEE Rev Biomed Eng.

[121] Kahankova R (2022). Review of recent advances and future developments in fetal phonocardiography. IEEE Rev Biomed Eng.

[122] Carolan M (2013). Gestational diabetes mellitus among women born in South East Asia: a review of the evidence. Midwifery.

[123] Chen D (2019). Deep learning and alternative learning strategies for retrospective real-world clinical data. npj Dig Med.

[124] Rasmy L (2021). Med-BERT: pretrained contextualized embeddings on large-scale structured electronic health records for disease prediction. npj Digit Med.

[125] Choi E (2016). RETAIN: An interpretable predictive model for healthcare using reverse rime attention mechanism. Adv Neural Inf Process Syst.

[126] Oliver C (2021). Longitudinal patient stratification of electronic health records with flexible adjustment for clinical outcomes. ML4H.

[127] Sharma P (2022). Data pre-processing using neural processes for modeling personalized vital-sign rime-series data. IEEE Journal of Biomedical and Health Informatics.

[128] Rajkomar A (2018). Scalable and accurate deep learning with electronic health records. NPJ Digital Med.

[129] Landi I (2020). Deep representation learning of electronic health records to unlock patient stratification at scale. npj Digit Med.

[130] Li L (2015). Identification of type 2 diabetes subgroups through topological analysis of patient similarity. Sci Transl Med.

